# Biophysical differences in IgG1 Fc-based therapeutics relate to their cellular handling, interaction with FcRn and plasma half-life

**DOI:** 10.1038/s42003-022-03787-x

**Published:** 2022-08-18

**Authors:** Torleif Tollefsrud Gjølberg, Rahel Frick, Simone Mester, Stian Foss, Algirdas Grevys, Lene Støkken Høydahl, Øystein Kalsnes Jørstad, Tilman Schlothauer, Inger Sandlie, Morten C. Moe, Jan Terje Andersen

**Affiliations:** 1 Department of Immunology, Oslo University Hospital, Rikshospitalet, 0372 Oslo, Norway; 2 Department of Pharmacology, Oslo University Hospital, University of Oslo, 0372 Oslo, Norway; 3 Department of Ophthalmology, Oslo University Hospital, University of Oslo, 0450 Oslo, Norway; 4 Department of Chemical and Biomolecular Engineering, Johns Hopkins University, Baltimore, MD USA; 5 KG Jebsen Coeliac Disease Research Centre, University of Oslo, 0372 Oslo, Norway; 6 Roche Pharma Research and Early Development (pRED), Roche Innovation Center Munich, 13, 82377 Penzberg, Germany; 7 Department of Biosciences, University of Oslo, 0316 Oslo, Norway

**Keywords:** Antibody therapy, Molecular medicine, Antibodies

## Abstract

Antibody-based therapeutics (ABTs) are used to treat a range of diseases. Most ABTs are either full-length IgG1 antibodies or fusions between for instance antigen (Ag)-binding receptor domains and the IgG1 Fc fragment. Interestingly, their plasma half-life varies considerably, which may relate to how they engage the neonatal Fc receptor (FcRn). As such, there is a need for an in-depth understanding of how different features of ABTs affect FcRn-binding and transport behavior. Here, we report on how FcRn-engagement of the IgG1 Fc fragment compare to clinically relevant IgGs and receptor domain Fc fusions, binding to VEGF or TNF-α. The results reveal FcRn-dependent intracellular accumulation of the Fc, which is in line with shorter plasma half-life than that of full-length IgG1 in human FcRn-expressing mice. Receptor domain fusion to the Fc increases its half-life, but not to the extent of IgG1. This is mirrored by a reduced cellular recycling capacity of the Fc-fusions. In addition, binding of cognate Ag to ABTs show that complexes of similar size undergo cellular transport at different rates, which could be explained by the biophysical properties of each ABT. Thus, the study provides knowledge that should guide tailoring of ABTs regarding optimal cellular sorting and plasma half-life.

## Abstract (web summary)

Analysis of clinically approved antibody-based therapeutics reveals different structural designs, such as full-length IgG1 or Fc-fusions, entail distinct biophysical properties that affect FcRn binding, intracellular transport and plasma half-life.

## Introduction

Most approved antibody-based therapeutics (ABTs) are of the immunoglobulin G (IgG) isotype, and in particular the IgG1 subclass^1,2^. In addition, the IgG1 fragment crystallizable (Fc) region is used as a fusion partner to prolong the circulatory half-life of therapeutic proteins^3–5^. Both these ABT modalities are used to treat a range of diseases, including ocular diseases, hemophilia, type 2 diabetes, autoimmunity and cancer. Fc-fusion partners include coagulation factors (eftrenonacog alfa, efmoroctocog alfa) and hormone analogs (romiplostim, dulaglutide) used for replacement therapy, as well as receptor domains (aflibercept, etanercept, abatacept, belatacept) used to block disease drivers, such as vascular endothelial growth factor (VEGF) and tumor necrosis factor (TNF)-α. Importantly, IgG1 and Fc-fusions may bind and neutralize the same soluble targets. Examples include the VEGF-binding aflibercept and bevacizumab^6,7^, and the TNF-α binding etanercept, infliximab and adalimumab^8^. Having multiple ABTs for a given indication is beneficial for life-long treatment, as the therapeutic effect of each may decrease over time due to immune responses raised against a specific ABT^9,10^. In such cases, efficacy may be restored by switching to an alternative ABT^11–14^.

Long plasma half-life prolongs dosing intervals, which increases treatment adherence^15,16^. However, while IgG1s have a half-life of 3 weeks on average, individual monoclonal IgG1s have strikingly different half-lives, ranging from 6 to 32 days^2,17^. In addition, most IgG1 Fc-fusions have considerably shorter half-lives than full-length IgG1s, exemplified by a mean half-life of below 5 days for etanercept^18^, eftrenonacog alfa^19^ and romiplostim^20^. While this may in part be explained by the Ag-sink effect, these large differences must be related to their biophysical properties, as the ABTs are administered in excess amounts.

In vivo plasma half-life of ABTs relates to engagement of the neonatal Fc receptor (FcRn), which acts as a homeostatic regulator^21,22^. FcRn is expressed throughout the body, where it rescues IgG and Fc-containing molecules from degradation in both hematopoietic and non-hematopoietic cells^23–27^. Specifically, the receptor predominantly resides in acidified endosomes, where it encounters IgG entering the cells by fluid-phase pinocytosis. FcRn binds IgG at pH 5.0–6.5, triggering recycling of the complex to the cell surface, where exposure to the neutral pH of the extracellular milieu results in ligand release^28–31^. As such, the FcRn-dependent rescue mechanism is pH-dependent. Strict pH-dependent binding also drives FcRn-mediated transcytosis across polarized epithelial cell layers^32,33^. In addition, FcRn may protect IgG-containing immune complexes^34,35^, or facilitate processing of immune complexes by various immune cells in concert with the classical Fcγ receptors^36–38^.

While the principle binding site for FcRn is located to the Fc region^39–41^, the Fab arms modulate binding, and potentially also interact directly with the receptor^42–46^. In addition, the biophysical properties of ABTs, such as isoelectric point (pI) and surface charge distribution, may modulate both target binding and plasma half-life^45,47–51^. This raises the need for thorough studies of how distinct biophysical properties of different ABT designs relate to their FcRn engagement, intracellular transport and plasma half-life. Hence, we here report on such a study, where we combine biochemical and cellular studies with structural analyses and in vivo studies in human FcRn (hFcRn)-expressing mice to gain insights into how different ABT designs with distinct biophysical properties engage FcRn, undergo intracellular transport and behave in vivo.

## Results

### An IgG1-derived Fc fragment exhibits short plasma half-life

**Fig. 1:**
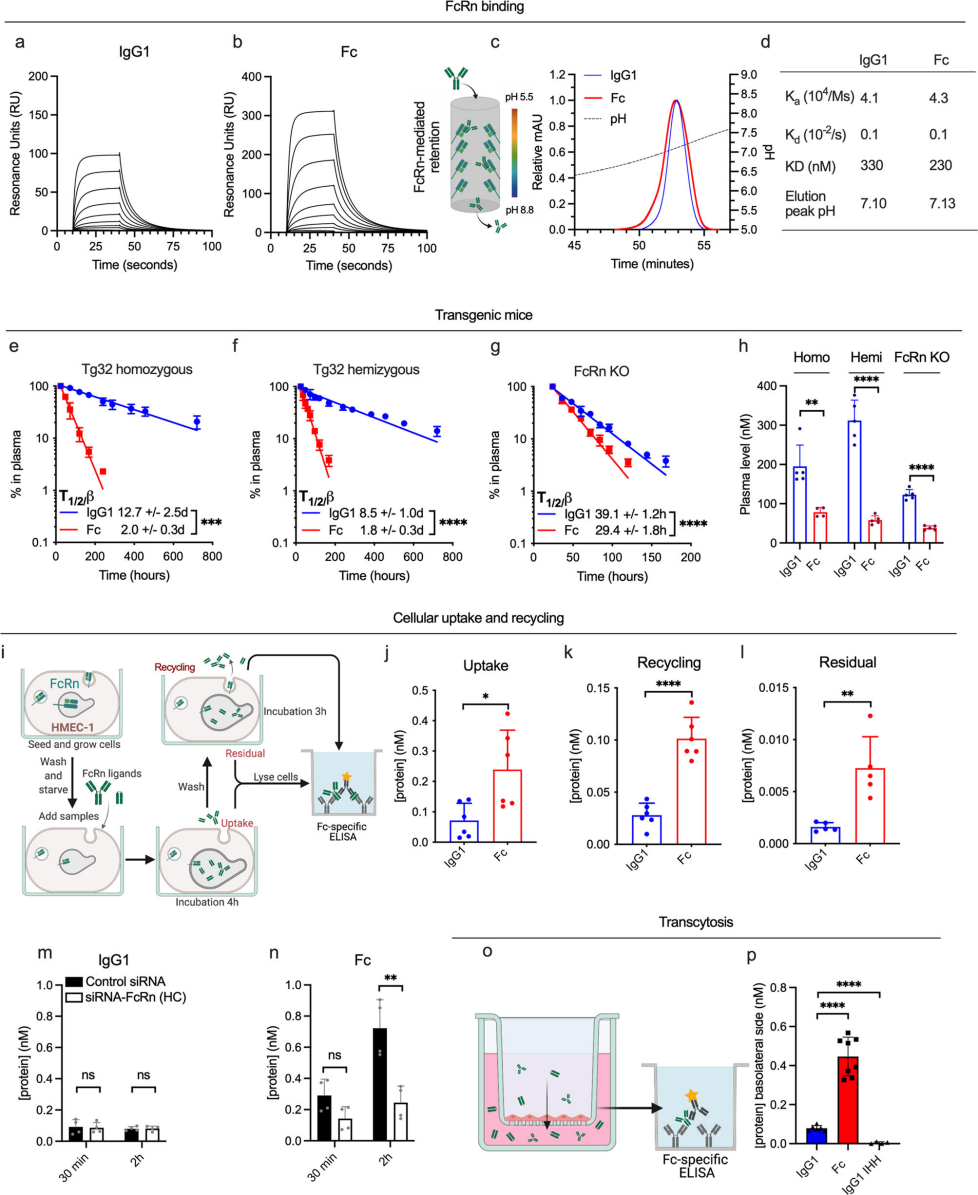
FcRn-mediated transport properties differ between the IgG1 Fc and full-length IgG1. **a**, **b** Representative SPR sensorgrams of titrated amounts of monomeric FcRn injected over anti-NIP IgG1 and the IgG1 Fc fragment (~300 RU). **c** FcRn affinity chromatography and resulting elution profiles of anti-NIP IgG1 (shown in blue) and the IgG1 Fc fragment (shown in red). **d** Table summarizing key parameters from FcRn interaction studies shown in **a**–**c**. Elimination curves and estimated β-phase half-life of anti-NIP IgG1 and IgG1 Fc fragment in **e** homozygous Tg32 mice, **f** hemizygous Tg32 mice, and **g** mice lacking FcRn (FcRn KO). **h** Molar amounts in plasma at the start of the β-phase (1 day after IV injection) in the same mice and order as displayed in **a**–**c**. *n* = 5 individual mice for all six groups. **i** Illustration of the HERA methodology. **j**–**l** HERA parameters obtained for anti-NIP IgG1 and the IgG1 Fc fragment. Shown data represents two independent, representative experiments. HERA uptake following siRNA knockdown of FcRn and varying incubation time for the **m** anti-NIP IgG1 and **n** the IgG1 Fc fragment. Shown data represents two independent, representative experiments. **o** Illustration of transcytosis methodology used to obtain data on apical to basolateral FcRn-dependent transcytosis in MDCK cells stably overexpressing hFcRn shown in **p**. Shown data represent two independent experiments. IgG1 IHH denotes IgG1 with the amino acid substitutions I253A, H310A and H435A to abolish FcRn binding. *p < 0.05, **p < 0.005, ***p < 0.0005, ****p < 0.0001 (two-tailed, unpaired Student’s *t* test). Data in bar plots show mean values ± SD.

We first used surface plasmon resonance (SPR) to compare FcRn-binding kinetics of a recombinantly produced IgG1-derived Fc fragment (50 kDa) and an IgG1 molecule (150 kDa), consisting of a human heavy chain paired with a mouse lambda light chain, with specificity for the hapten 4-hydroxy-3-iodo-5-nitrophenylacetic acid (NIP)^52,53^. IgG1 and Fc proteins were immobilized (≈400 RU), followed by injection of titrated amounts of monomeric receptor at pH 5.5. The obtained sensorgrams (Fig. 1a, b) were fitted to a 1:1 Langmuir binding model and yielded similar dissociation constants (KD) of 330 and 230 nM for IgG1 and the Fc fragment, respectively (Fig. 1d). Next, the proteins were injected on an FcRn-coupled affinity chromatography column at pH 5.5 (Fig. 1c), followed by elution with a gradual increase of pH to 8.8 to trigger receptor release^54^. This was done to mimic the endosomal pH-gradient, where FcRn engages IgG1 at acidic pH and releases it as the pH approaches neutral. Overlapping elution profiles were observed, with peaks at pH 7.11 and 7.13 for IgG1 and the Fc fragment, respectively.

To address how presence of the Fab arms affects plasma half-life, equimolar amounts of the proteins were intravenously injected into two distinct transgenic mouse strains expressing hFcRn, but at different levels, while lacking the mouse counterpart, namely homozygous and hemizygous Tg32 mice (Fig. 1e, f, h). Blood samples were collected for up to 30 days and plasma levels quantified by a two-way Fc-based ELISA. This revealed a 6.3-fold difference in half-life in Tg32 homozygous mice, corresponding to 12.7 days for IgG1 and 2.0 days for the Fc fragment. As expected, the half-lives were shorter in Tg32 hemizygous mice (8.5 vs. 1.8 days), but still, a 4.6-fold difference was measured. To assess the role of FcRn, the experiment was repeated in mice lacking FcRn (FcRn KO). Here, the half-life was only 39.1 h for IgG1, 7.8-fold shorter than that in Tg32 homozygous mice. However, the half-life was still 1.3-fold longer for full-length IgG1 than for the Fc fragment (Fig. 1g). Comparison of plasma concentrations 1 day after injection revealed that the elimination rate of the Fc fragment was higher than that of the IgG1 (Fig. 1h).

For further pharmacokinetic assessment, the serum concentrations of Fc and IgG1 were fitted to a non-compartmental pharmacokinetic model in MatLab^55^. Resulting values are listed in Supplementary Table 1. Fc had a higher clearance rate and volume of distribution than IgG1 in all three mouse models, likely due to its lower molecular weight. In Tg32 homozygous mice, the clearance of Fc was ~15-fold higher than that of IgG1, while in FcRn KO mice, this difference was reduced to 3.6-fold. Importantly, the Fc clearance in FcRn KO mice was 2.2-fold higher than in Tg32 homozygous mice, demonstrating that the Fc is also protected by FcRn-dependent mechanisms. Of note, the half-lives calculated by this method indicate a larger difference between the two molecules than what we observed experimentally.

Thus, despite the fact that FcRn binding kinetics, as assessed by SPR, and pH-dependent dissociation from FcRn, as assessed by chromatography, is similar for the two molecules, their serum half-lives were very different. The difference is partly FcRn dependent and partly determined by characteristics such as size and volume of distribution.

### IgG1 and the Fc fragment are handled differently by cells

To compare the two molecules in a cellular context, we used a human endothelial cell-based recycling assay (HERA)^56^ (Fig. 1i), which takes advantage of an adherent human endothelial cell line stably overexpressing hFcRn^57^. Equimolar amounts of IgG1 and the Fc fragment were added to the cells followed by incubation for 3 h to allow for uptake. Next, the cells were washed and either lysed or placed in IgG-depleted growth medium, to allow for detection of cellular uptake, recycling or retention after additional 3 h, when the medium was collected and the cells washed and lysed. The two-way anti-Fc ELISA used for quantification demonstrated that 3.4-fold more of the Fc fragment than the IgG1 entered the cells during the uptake step (Fig. 1j), 5.3-fold more of the Fc was detected in the recycling medium (Fig. 1k), and 5.4-fold more had also accumulated inside cells at the termination of the assay (Fig. 1l).

To gain further insight into the uptake mechanism, we compared the amounts taken up after incubation for 30 min and 2 h, which revealed that 2.2- and 7.1-fold more Fc was taken up relative to IgG1 after the respective incubation times (Fig. 1m, n). Next, we addressed the FcRn contribution by treating the cells with siRNA targeting the FcRn heavy chain, using an established protocol^56^. Almost equal intracellular amounts of the two were measured after 30 min, and after 2 h, only 2.4-fold more of the Fc was detected. Thus, while the cellular uptake of the Fc fragment was largely dependent on FcRn (Fig. 1n), the uptake of the IgG1 was less so (Fig. 1m). To further study FcRn-dependent uptake of IgG1, we included an anti-NIP IgG1 Fc-engineered variant with five amino acid substitutions (M252Y/S254T/T256E/H433K/N434F; MST/HN) that improve FcRn-binding at both acidic and neutral pH, thereby enabling FcRn engagement at the cell surface^53,58^. As expected, this variant displayed an increased uptake relative to non-engineered IgG1, at a magnitude of 15-fold after 2 h incubation, and siRNA-treatment reduced its uptake by 4.7-fold (Supplementary Fig. 1a).

As FcRn also mediates transcytosis, we compared transcellular transport of the two molecules using a transwell system seeded with a Madin-Darby Canine Kidney (MDCK) cell line stably transfected with hFcRn (Fig. 1o). The proteins were added to the apical side and samples collected at the basolateral side 4 h later. Quantification by the two-way ELISA revealed 5.5-fold more of the Fc than IgG1 in the basolateral chamber (Fig. 1p). An anti-NIP IgG1 harboring three Fc amino acid substitutions that abolish receptor binding (I253A/H310A/H435A; IHH^30,34^) hardly reached the basolateral side (Fig. 1p).

In summary, the Fc fragment is internalized, recycled and transcytosed efficiently by an FcRn-dependent mechanism. In comparison, these mechanisms are less efficient for IgG1. Importantly, despite the high uptake and recycling of the Fc fragment, there is still a substantial residual intracellular fraction, resulting in degradation that likely contributes to its shorter half-life.

### The nature of the Fc fusion partner affects pharmacokinetics

**Fig. 2:**
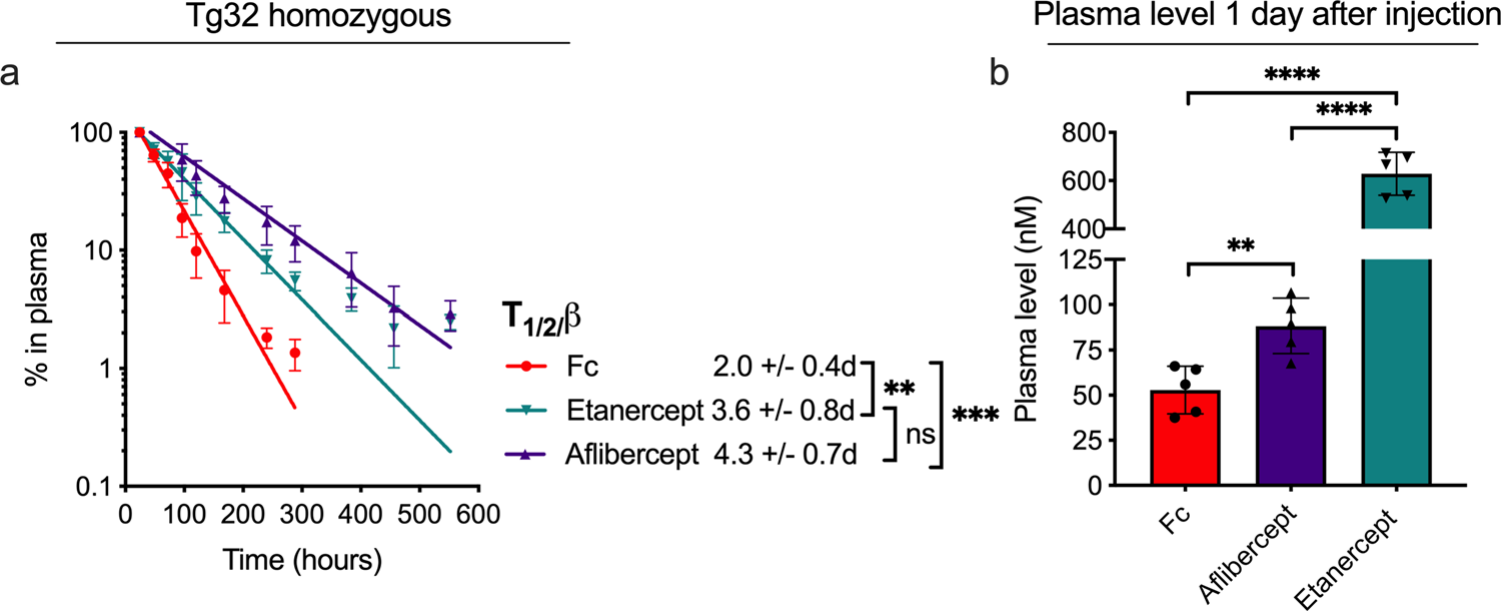
Circulatory properties of IgG1 Fc and Fc-fusions in hFcRn transgenic mice. **a** Elimination curves and estimated β-phase half-life of the IgG1 Fc fragment (shown in red), aflibercept (shown in dark purple) and etanercept (shown in teal) in homozygous Tg32 mice. **b** Molar amounts at the start of the β-phase (1 day after IV injection). *n* = 5 individual mice per group. **p < 0.005, ***p < 0.0005, ****p < 0.0001 (two-tailed, unpaired Student’s *t* test). Data show mean values ± SD.

To investigate the effect of adding fusion partners other than anti-NIP Fab to the Fc region, we studied two ABTs with Fc-fused Ag-binding receptor domains, namely etanercept and aflibercept. Specifically, etanercept (MW ≈ 103 kDa) consists of the extracellular domains of TNF receptor 2 (TNFR2) fused to the IgG1 Fc, while aflibercept (MW ≈ 97 kDa) consists of the extracellular domain 2 of VEGF receptor (VEGFR) 1 linked to domain 3 of VEGFR2, creating a joint receptor domain fused to the IgG1 Fc which can bind both VEGF and placental growth factor^59^. First, plasma half-life was determined in Tg32 homozygote mice (Fig. 2a), where etanercept (3.6 days) and aflibercept (4.3 days) showed 1.8- and 2.2-fold extended half-life, respectively, compared to the Fc fragment (2.0 days). Furthermore, comparing plasma concentrations after 1 day revealed 12-fold more of etanercept than the Fc, but only 1.7-fold more of aflibercept (Fig. 2b). Additional pharmacokinetic parameters are listed in Supplementary Table 2, and show a maximum serum concentration of etanercept 26.8-fold higher than that of the Fc (64.4 vs. 2.4 µg/ml, respectively). Notably, the approximate two-fold longer half-life of these Fc-fusions compared to the IgG1 Fc could be due to their higher MW.

To address whether size could explain the longer half-life, we performed a similar comparison of IgG1 Fc and an additional Fc-fusion ABT with MW in the same range as aflibercept and etanercept, namely eftrenonacog alfa (MW ≈ 98 kDa), which consists of human coagulation factor IX fused to the IgG1 Fc (Supplementary Fig. 2). HERA parameters (Supplementary Fig. 2a–c) revealed this Fc-fusion to have an uptake roughly 3-fold increased relative to the Fc (Supplementary Fig. 2a), but recycling in the same range as the anti-NIP IgG1 (Supplementary Fig. 2b). This was reflected by the Fc-fusion displaying residual amounts nearly 3-fold that of the IgG1 Fc (Supplementary Fig. 2c), suggesting that the fusion partner confer increased cellular retention. Conversely, eftrenonacog alfa displayed circulatory properties near identical to that of the IgG1 Fc in Tg32 homozygous mice (Supplementary Fig. 2d, e).

**Fig. 3:**
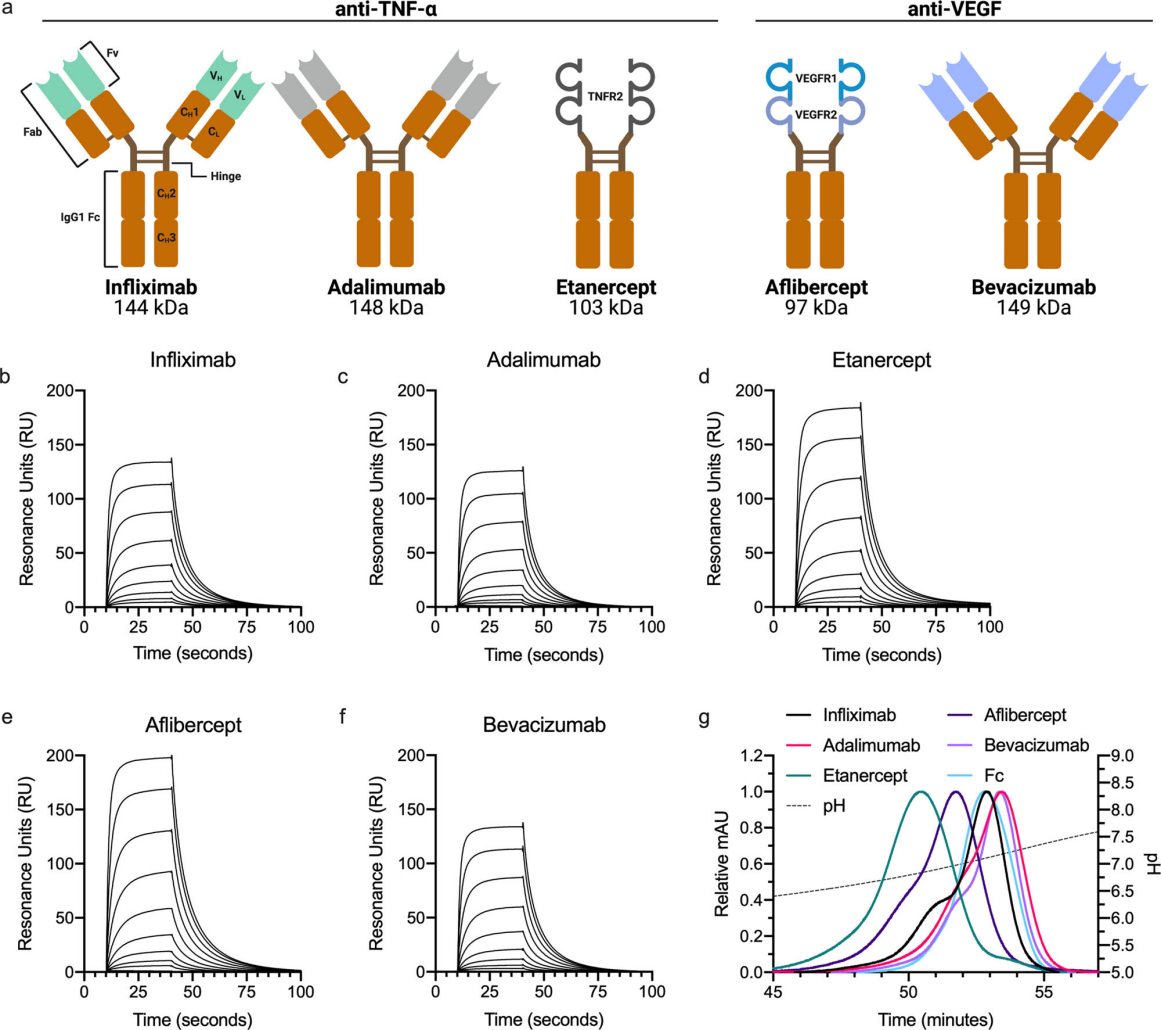
Fc-fused modalities affect FcRn-binding throughout the pH-gradient. **a** Schematic illustration of structural elements in the ABT panel. All five ABTs contain an IgG1 Fc (orange, CH2 and CH3). ABMs (Fvs and receptor domains) are marked in light green, gray or shades of blue. Infliximab is a chimeric mouse-derived human IgG1 with a murine Fv (marked in light green) binding to TNF-a. Adalimumab is a fully human IgG1 binding to TNF-a (Fv gray). Etanercept consists of the extracellular domains of TNFR2 (gray) fused to the IgG1 Fc via a hinge region. Aflibercept consists of D2 of VEGFR1 (turquoise) linked to D3 of VEGFR2 (light blue) fused to an IgG1 Fc. Bevacizumab is a fully human IgG1 binding to active isotypes of VEGF (Fv light blue). MW of the total amino acid sequence of each ABT is indicated. **b**–**f** Representative SPR sensorgrams of titrated amounts of monomeric FcRn injected over immobilized ABTs (~400 RU). **g** Elution profiles of ABTs from FcRn chromatography shown as relative fluorescence intensity throughout the pH gradient. The pH is plotted on the right *Y*-axis and indicated by a stapled line. Color labeling; infliximab (black), adalimumab (pink), etanercept (teal), aflibercept (dark purple), bevacizumab (light purple), Fc (light blue).

Next, we compared the Fc-fusions with three full-length IgG1 variants, namely the anti-TNF-α IgG1s infliximab and adalimumab, as well as the anti-VEGF IgG1 bevacizumab. The IgG1s have clinical half-lives distinct from that of the Fc-fusions (ranging from 2–4 days for etanercept^18^ to ~20 days for bevacizumab^60^; summarized in Table 1). A schematic overview of their structural elements is shown in Fig. 3a. Despite some minor variations in amino acid sequence, highlighted in Supplementary Fig. 3e, both the Fc-fusions and the IgG1s contain the same Fc. Thus, the main variable is their Ag-binding moieties (ABMs) and the way they bind their cognate Ags (visualized in Supplementary Fig. 3a). While TNF-α forms a trimer in solution, VEGF is a dimer^61,62^. Solved crystal structures of each ABM, defined as fragment variables (Fvs) for IgG1s and receptor domains for Fc-fusions, are available through the Protein Data Bank (PDB), and are shown in Supplementary Fig. 4a. Of note, as no X-ray crystallographic structure of the aflibercept ABM has been solved, we visualized it by retrieving the structures of both its receptor domains and modeling their likely conformation by the use of Rosetta.

**Table 1:** Clinical half-lives, charge parameters and FcRn-binding capacities of ABTs and their cognate Ags.

		ABM charge		AIR charge		Ag charge		FcRn binding			
ABT	Clinical *t*_1/2_ (days)^a^	pH 5.5	pH 7.4	pH 5.5	pH 7.4	pH 5.5	pH 7.4	*K*_a_(10^4^/Ms)	*K*_d_ (10^−2^/s	KD (nM)	Elution peak pH
Infliximab	8–10	3.1	−1.6	3.7	1.3	TNF-α		3.5	0.2	310	7.15
Adalimumab	14 (10–20)	5.2	2.2	2.0	1.1	−6.1	−15.4	4.2	0.1	430	7.21
Etanercept	2–4	3.2	0.8	3.1	3.0			3.1	0.1	380	6.88
Aflibercept	5–6	10.8	4.3	5.9	4.2	VEGF		3.0	0.1	350	7.01
Bevacizumab	20 (10–50)	5.0	1.4	−0.3	−2.7	−2.1	−10.0	3.2	0.1	380	7.20

^a^The stated clinical plasma half-lives of ABTs were retrieved from FDA package inserts. For bevacizumab, the stated clinical half-life of 20 days refers to a large, clinical study following intravenous injection^60^.

First, SPR-derived FcRn-binding kinetics of the ABTs at pH 5.5 were measured, which revealed KD values for all five in the range of 310–434 nM (Fig. 3b–f and Table 1). All eluted from the FcRn affinity chromatography column with peaks ranging from pH 6.88 to 7.21 (Fig. 3g and Table 1). Specifically, the Fc-fusions eluted before the IgG1s, and etanercept (6.88) before aflibercept (7.01). The IgG1s eluted from pH 7.15 (infliximab) to 7.20–7.21 (adalimumab and bevacizumab). Thus, the large differences in plasma half-lives observed for the five ABTs could not be predicted by differences observed in FcRn binding detectable by SPR or release from the receptor-coupled column.

### IgG1s and Fc-fusions have distinct charge characteristics

Differences in cellular handling may be due to charge characteristics. In general, net positive charge may cause unspecific interactions with negatively charged cell membranes and off-target binding, while positive surface charge in Ag-interacting residues (AIRs) may delay release from FcRn^45^. Importantly, such patches may be partially or completely masked upon Ag-binding, which may change the overall charge of the immune complex. Last, but not least, the net charge of ABMs and AIRs may change as a function of pH. Net charge characteristics of the five ABTs and their cognate Ags at pH 5.5 and 7.4 are summarized in Table 1, and surface charge distribution and net charge of their ABMs throughout the endosomal pH-gradient are visualized in Supplementary Fig. 4b–f. For IgG1s, we determined AIRs as complementarity determining regions (CDRs). For receptor domains, we determined AIRs as amino acid residues within 5 Å of bound Ag in solved receptor domain-Ag co-crystal structures. Net and surface charge of VEGF and TNF-α are shown in Supplementary Fig. 3b–d.

These analyses show that the ABMs of both anti-TNF-α IgG1s display a positive charge at pH 5.5, as does that of the TNF-α receptor domain-fusion. In the AIRs, this positive charge is somewhat pH-dependent, but only for the IgG1s. TNF-α in itself is negatively charged at both pH 5.5 (−6.1) and 7.4 (−15.4), at a magnitude that ensures large net negative charge of all three anti-TNF-α ABMs after Ag-binding, regardless of pH.

The ABMs of the anti-VEGF IgG1 and VEGF receptor domain-fusion are also positively charged throughout the pH-gradient, and the VEGF receptor domain is more so than the other ABMs. In the anti-VEGF IgG1, AIRs are negatively charged at both pH 5.5 (−0.3) and 7.4 (−2.7), while the VEGF receptor domain of aflibercept has positively charged AIRs at both pH 5.5 (5.9) and 7.4 (4.2). As the net charge of VEGF at pH 5.5 is −2.1, The VEGF receptor domain will be net positively charged also after Ag-binding, while the corresponding anti-VEGF IgG1 immune complex will have negative charge. At pH 7.4, the net VEGF charge is −10.0, and both anti-VEGF IgG1 and receptor domain-fusion immune complexes will have net negative charge.

### Fusion partner characteristics and pH affect Ag-binding and complex formation

**Fig. 4:**
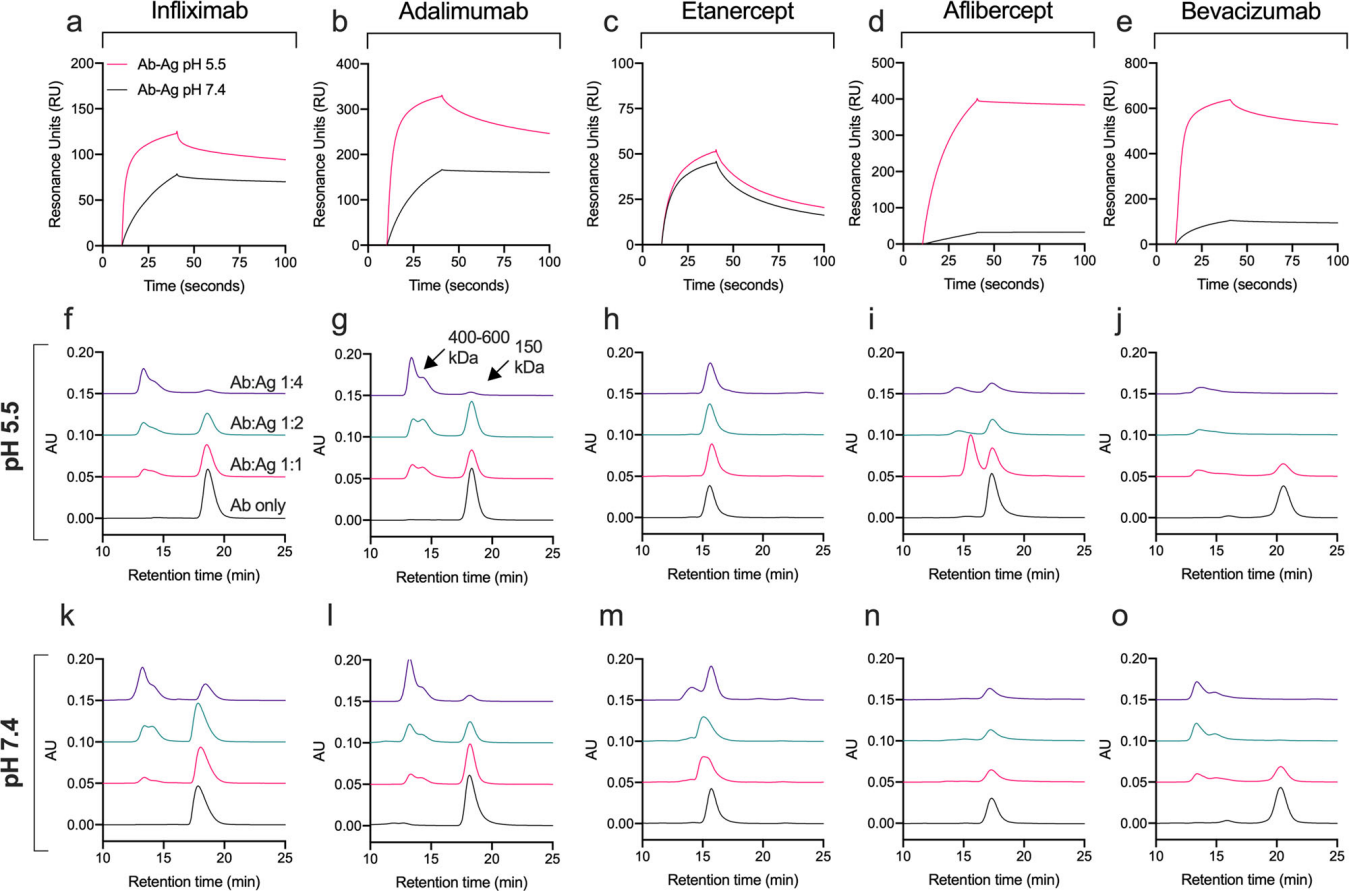
Ag-binding and immune complex formation are affected by pH and differ between IgG1s and Fc-fusions. **a**–**e** SPR sensorgrams showing ABTs (100 nM) injected over and binding to immobilized cognate Ags (~300 RU) at either pH 5.5 or 7.4. Analytical SEC of ABTs preincubated with increasing amounts of their cognate Ag at either (**f**–**j**) pH 5.5 or (**k**–**o**) 7.4 before injection. Color labeling **a**–**d**; Ab-Ag injected at pH 5.5 (red) and at pH 7.4 (black). Color labeling **f**–**o**; Ab only (black), Ab:Ag 1:1 (red), Ab:Ag 1:2 (teal), Ab:Ag 1:4 (dark purple).

In a clinical setting, the ABTs will bind and neutralize the activity of soluble Ags. Due to differences in the pH-dependent charge in their AIRs, we suspected that pH could affect their Ag binding differently. To address this, all five ABTs were injected over immobilized TNF-α or VEGF in SPR at pH 5.5 and pH 7.4. Acidic pH increased the on-rate of both the anti-TNF-α IgG1s relative to that observed at pH 7.4 (Fig. 4a, b), but not of the anti-TNF-α receptor domain-fusion (Fig. 4c). For VEGF, acidic pH enhanced the on-rate more than 5-fold of both the anti-VEGF IgG1 and anti-VEGF receptor domain-fusion (Fig. 4d, e). Thus, all ABTs with pH-dependent reduction of positive charge in the AIRs (Table 1) exhibited increased Ag-binding at acidic pH.

The size of the immune complexes resulting from ABTs binding to their cognate Ags is determined by the monomeric or multimeric state for the Ag, the availability of Ag-binding sites, and the properties of the ABT. We next addressed how the five ABTs formed complexes with their cognate Ags in solution. All were incubated with increasing concentration of the Ags at pH 5.5 and at pH 7.4, prior to injection on a high-resolution analytical size exclusion chromatography (SEC) column. We first observed the ABTs eluting as monomeric peaks with slightly different retention times in the absence of Ag. Then, in the presence of Ags, the three IgG1s formed large immune complexess that eluted earlier than the monomers at both pH conditions (Fig. 4f, g, j–l, o). At a 4-fold molar excess of Ag, multimerization was observed for all IgG1s.

In stark contrast, the two Fc-fusions exhibited only minor elution shifts in the presence of cognate Ag (Fig. 4h, i, m, n). For the anti-TNF-α receptor domain-fusion, heterogenous complex formation was observed at four-fold excess of Ag at pH 7.4, suggesting a mix of 1:1 and 1:2 complexes (Fig. 4m). For the anti-VEGF receptor domain-fusion, two distinct peaks were observed at pH 5.5 (Fig. 4i), but not at pH 7.4 (Fig. 4n). Thus, the IgG1s and Fc-fusions engaged their cognate Ags very differently, in that only the IgG1s formed large, multimeric immune complexes.

### Ag binding alters binding to FcRn

**Fig. 5:**
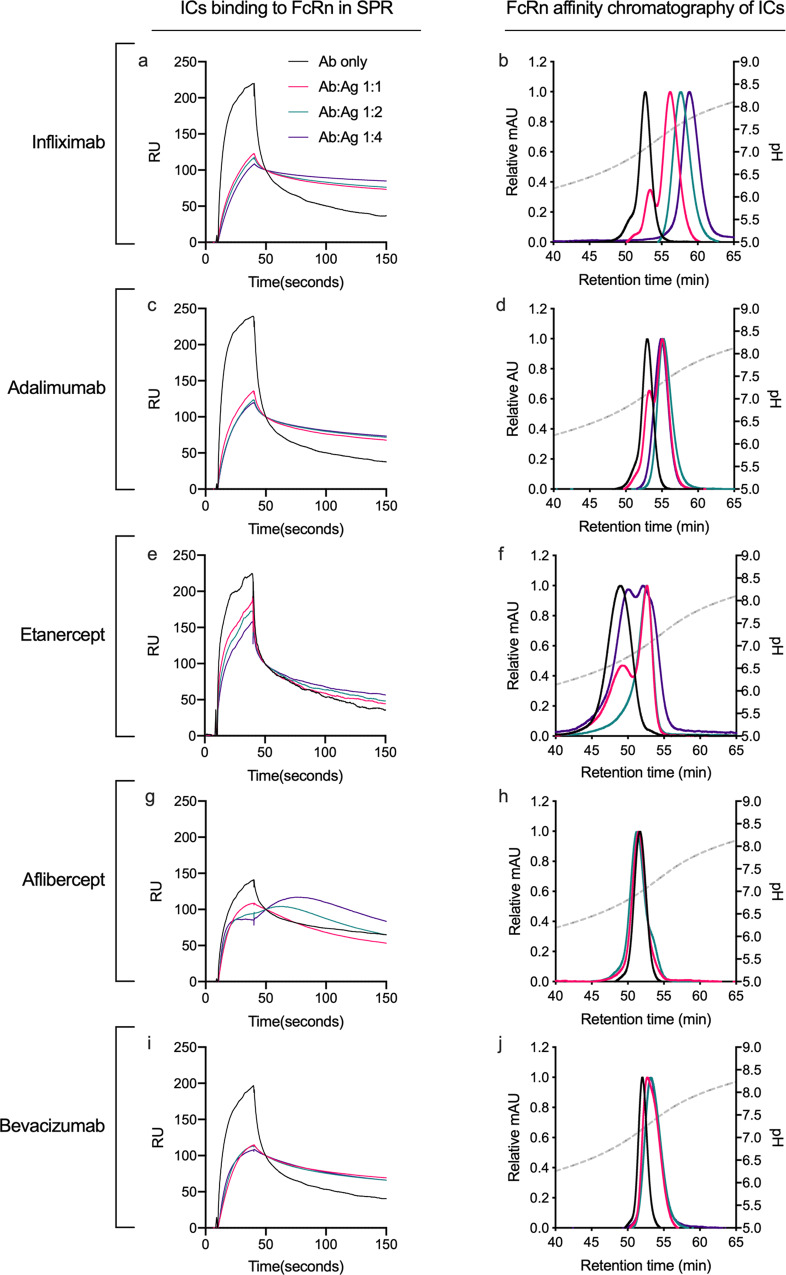
Ag-binding alters the Fc-FcRn interaction. **a**, **c**, **e**, **g**, **i** Sensorgrams showing injection of 15.62 nM ABTs preincubated with increasing amounts of cognate Ags at pH 5.5 and injected over immobilized (~100 RU) site-specifically biotinylated FcRn at pH 5.5. **b**, **d**, **f**, **h**, **j** Elution profiles from FcRn chromatography of ABTs preincubated with increasing amounts of cognate Ags at pH 5.5 shown as relative fluorescence intensity and as a function of pH (plotted on the right *Y*-axis, indicated by a stapled line) Color labeling; Ab only (black), Ab:Ag 1:1 (red), Ab:Ag 1:2 (teal), Ab:Ag 1:4 (dark purple).

To address FcRn-binding of Ag-bound ABTs, ABTs were preincubated with increasing concentrations of TNF-α or VEGF and injected over immobilized FcRn at pH 5.5 in SPR (Fig. 5a, c, e, g, i). The results in all cases showed FcRn binding in the presence of Ag, but with reduced peak responses and slower dissociation rates than for naked ABTs. This was more pronounced for the large IgG1 immune complexes (Fig. 5a, c, i) than the smaller Fc-fusion equivalents (Fig. 5e, g).

When Ag-bound ABTs were analyzed by analytical FcRn affinity chromatography, all three TNF-α-bound immune complexes again showed prolonged retention times (Fig. 5b, d, f). In comparison, a similar shift could not be detected upon VEGF binding (Fig. 5h, j). Thus, whether or not Ag-binding affects the pH-dependent dissociation from FcRn, does not depend on the size of the immune complexes formed, but rather their content.

### Distinct differences in FcRn-mediated recycling of ABTs and immune complexes

**Fig. 6:**
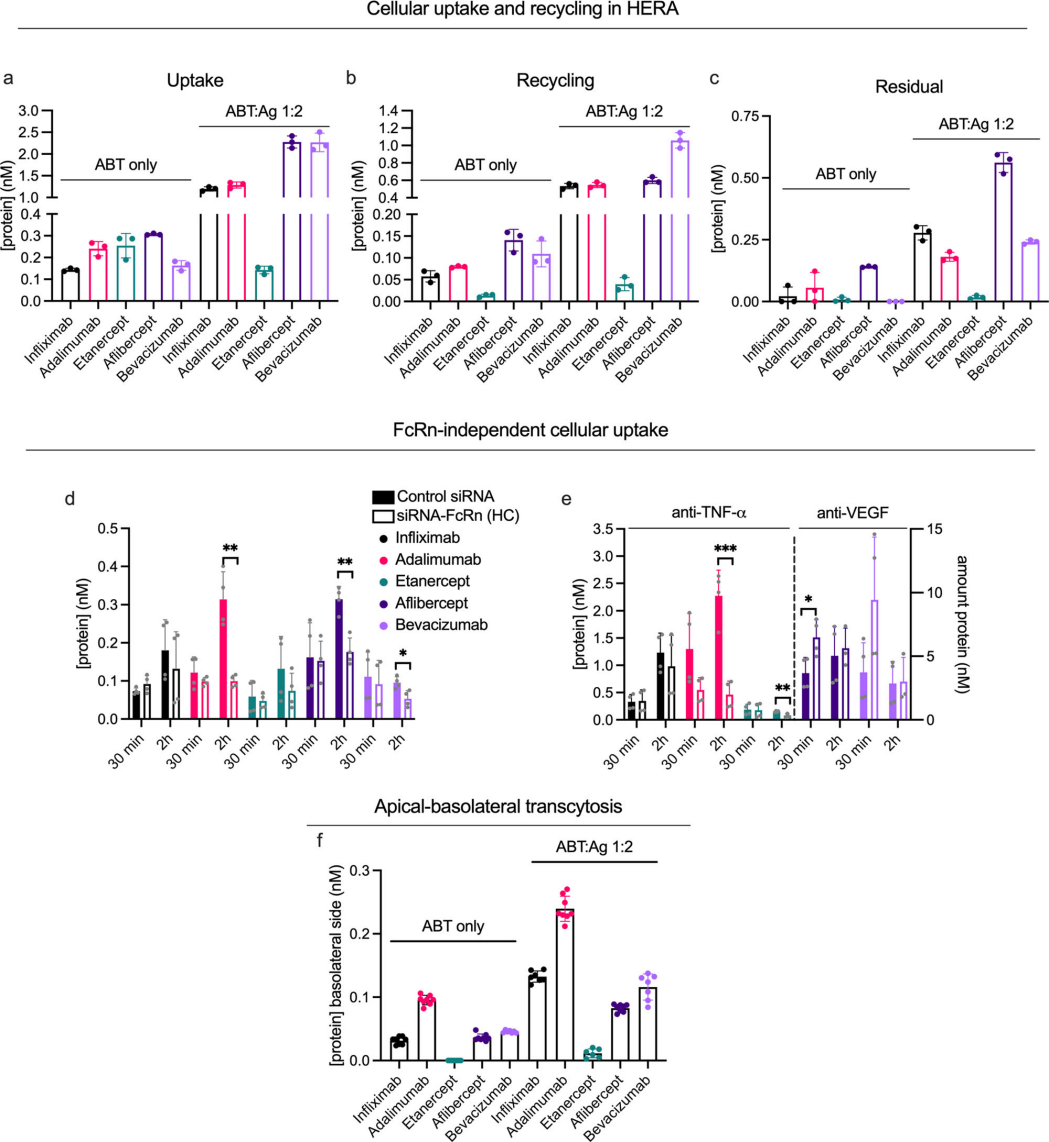
FcRn-mediated cellular handling differs between ABTs with distinct Fc-fused modalities. **a**, **c**, **e** HERA parameters from one representative experiment, assessing amounts of ABTs as monomers (ABT only) and preincubated with a two-fold molar excess of their cognate Ags at pH 7.4 (ABT:Ag 1:2) taken up (**a**), recycled by (**b**) and kept in (**c**) HMEC-1-FcRn cells (*n* = 3 per bar). Two representative experiments showing amount of ABTs, as both monomers (**d**) and after preincubation with two-fold molar excess of cognate Ags (**e**), taken up after incubation for 30 min or 2 h on HMEC-1-FcRn cells treated with control siRNA or siRNA against the HC of FcRn. In **e**, anti-TNF-α and anti-VEGF ABTs are plotted on the left and right *y*-axis, respectively, as indicated. Differences between ctrl and siRNA-FcRn-treated cells for each sample group are indicated (*p < 0.05, **p < 0.005, ***p < 0.0005, two-tailed, unpaired Student’s *t* test). *n* = 4 per bar. **f** Amount of monomeric and Ag-bound ABTs on the basolateral side of polarized MDCK-hFcRn cells following apical application and an incubation of 4 h. Values correspond to two independent experiments (*n* = 8 per bar). Color labeling; infliximab (black), adalimumab (pink), etanercept (teal), aflibercept (dark purple), bevacizumab (light purple). Data show mean values ± SD.

To assess cellular handling of the ABTs and their immune complexes, we performed HERA, followed by detection in the two-way Fc-based ELISA. We found the two anti-TNF-α IgG1s and the anti-TNF-α receptor domain-fusion to have similar uptake efficiency in the absence of Ag (Fig. 6a). However, recycling of the IgG1s was 4-5-fold more efficient than that of the receptor domain-fusion (Fig. 6b). Comparing the anti-VEGF IgG1 and receptor domain-fusion revealed a two-fold higher uptake and a 1.3-fold higher recycling of the receptor domain-fusion (Fig. 6a, b). However, the residual amount of the anti-VEGF receptor domain-fusion was also high and its subsequent intracellular degradation will likely contribute to the short plasma half-life (Fig. 6c). Very little residual bevacizumab was observed. Thus, neither of the two receptor domain-fusions were as efficiently recycled as the IgG1s with corresponding specificities.

Next, prior to studying transport of Ag-bound ABTs, we addressed if adding TNF-α and VEGF to HERA affected cellular uptake and recycling. We mixed the anti-VEGF IgG1 with TNF-α at a 2-fold molar excess, and also one of the anti-TNF-α IgG1s together with VEGF (Supplementary Fig. 1c, d). As the results revealed no major impact of free Ag, the effect of immune complex formation was studied, by adding each of the five ABTs after preincubation with a two-fold molar excess of specific Ag. A summary of the resulting findings is shown in Table 2.

**Table 2:** Summary of key cellular transport parameters of ABTs in the absence and presence of cognate Ag.

	Uptake^a^		Recycling^a^		Relative effect of Ag binding			
ABT	pH 6.0	pH 7.4	pH 6.0	pH 7.4	Uptake	Recycling	Residual	Transcytosis
Infliximab	1.5	1.1	1.2	0.5	8.4	9.3	12.8	4.2
Adalimumab	1.7	1.6	1.7	1.1	5.3	6.9	3.2	2.5
Etanercept	0.2	0.4	0.1	0.2	0.6	3.0	2.1	NA
Aflibercept	4.2	6.7	0.9	1.3	7.4	4.3	3.9	2.5
Bevacizumab	1.3	0.8	0.8	0.4	13.9	9.7	NA	2.3

^a^Calculated as relative to anti-NIP IgG1 in the absence of competition, i.e., data shown in Fig. 7b, d.

For the anti-TNF-α IgG1s, Ag-binding increased uptake by five-fold (Fig. 6a) and recycling more than seven-fold (Fig. 6b). In contrast, Ag-binding increased neither uptake nor recycling of the anti-TNF-α receptor domain-fusion (Fig. 6a). Thus, binding to TNF-α enhanced uptake, and subsequent recycling, of ABTs displaying (1) large immune complex formation and (2) a pH-dependent charge in AIRs. For the anti-VEGF IgG1 bevacizumab, Ag-binding resulted in a 14-fold increase in uptake (Fig. 6a), and a 9-fold increase in recycling (Fig. 6b). In comparison, Ag-binding yielded 7-fold increase in uptake and a 4-fold increase in recycling for the anti-VEGF receptor domain-fusion. Importantly, at termination of the assay, 2.3-fold more of VEGF-bound receptor domain-fusion than VEGF-bound IgG1 had accumulated intracellularly (Fig. 6c).

To study how the biophysical properties of ABTs and Ags affect cellular uptake independently of FcRn, we measured uptake after 2 h incubation in cells treated with FcRn-targeting siRNA in HERA. We added ABTs to cells in the absence (Fig. 6d) and presence (Fig. 6e) of Ag. We found the effect of FcRn-knockdown to vary somewhat between the ABTs, but the intracellular levels of all ABTs tended to decrease in the absence of Ag (Fig. 6d). Then, in the presence of Ag, uptake of only one of three IgG1s significantly dropped after siRNA treatment (adalimumab; Fig. 6e). Thus, immune complex size was not the determining factor. For the receptor domain-fusions, the effect of FcRn-knockdown differed, as the intracellular level of Ag-bound anti-TNF-α receptor domain-fusion decreased, while that of the anti-VEGF receptor domain-fusion was unaffected (Fig. 6e).

### Fusion partners affect FcRn-mediated transcytosis

We next studied the ABTs in the MDCK-FcRn transwell system, and found both anti-TNF-α IgG1s to be transcytosed at rates higher than that of the anti-TNF-α Fc-fusion (Fig. 6f). Notably, the anti-TNF-α IgG1s differed, as the amounts of transcytosed adalimumab were 3.5-fold higher than infliximab. The anti-VEGF IgG1 and the corresponding Fc-fusion were transcytosed well and at similar levels.

Next, as VEGF and TNF-α are potent permeability factors^63,64^, we confirmed that neither transport of IgG1 nor transepithelial electrical resistance were drastically affected by adding the Ags to the medium (Supplementary Fig. 1h, i). This allowed studies of how immune complex formation affected transcellular transport in the MDCK-FcRn transwell system, and we found 2- to 4-fold increased transcytosis for all ABTs in the presence of their cognate Ags. In stark contrast to HERA, the hierarchy between the ABTs was largely unaffected by Ag-binding. Thus, the effect of Ag-binding on transcytosis and cellular recycling were distinct.

### Varying pH and competition for FcRn-binding reveal distinct recycling capacities of Fc-fusions

**Fig. 7:**
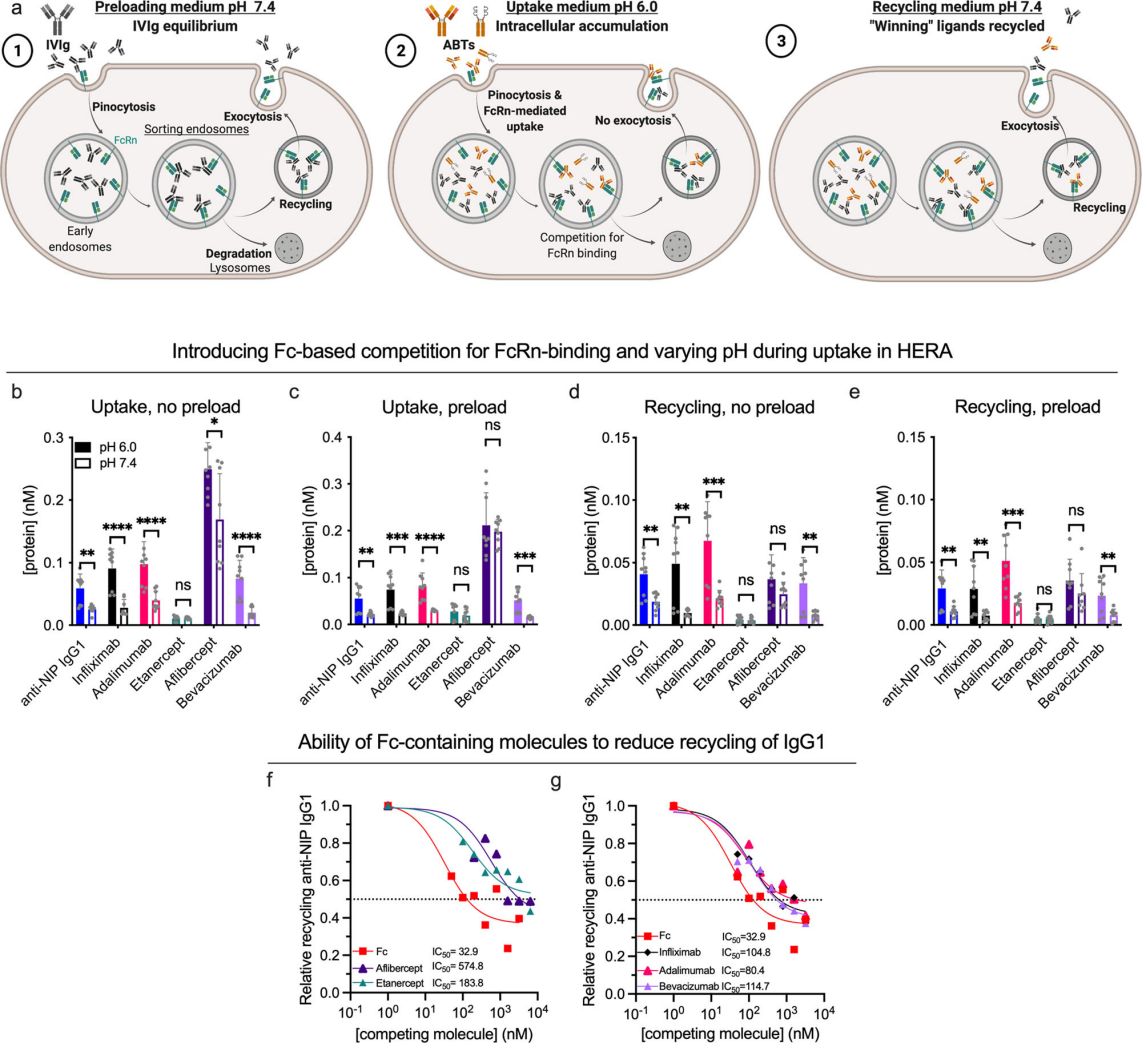
Varying pH and introducing competition for cellular recycling affect cellular handling of ABTs differently. **a** Illustration of the HERA competition protocol. Initially, cells are preloaded for 1 h with 7.5 mg/ml IVIg at pH 7.4 (1). Next, the uptake phase is conducted at either pH 7.4 or pH 6.0 to force intracellular accumulation (2), followed by (3) adding of recycling medium (pH 7.4) and a 3-h incubation. **b**, **c** Three experiments showing amount of ABTs detected in cells at the end of an uptake phase of either pH 6.0 (solid bars) or pH 7.4 (hollow bars) in either the absence (no preload, **b**) or presence (preload, **c**) of competition. *n* = 9 per bar, arising from three individual experiments. Differences between values measured after uptake at pH 6.0 and pH 7.4 are indicated (*p < 0.05, **p < 0.005, ***p < 0.0005, ****p < 0.0001, two-tailed, unpaired Student’s *t* test). **d**, **e** Recycled ABT amounts in either the absence (**d**) or presence (**e**) of competition, following uptake at either pH 6.0 or 7.4. **f**, **g** Amount of recycled anti-NIP IgG1 following simultaneous addition of titrated amounts of ABTs or the IgG1 Fc fragment and 400 nM anti-NIP IgG1 at pH 6.0 to preloaded cells. Values correspond to one representative experiment (*n* = 3 per data point). Color labeling; anti-NIP IgG1 (blue), infliximab (black), adalimumab (pink), etanercept (teal), aflibercept (dark purple), bevacizumab (light purple), Fc (red). Data in bar plots show mean values ± SD.

Injected ABTs will compete with endogenous IgG for binding to FcRn. However, due to the absence of immunogenic stimuli in pathogen-free housing and the poor interaction between mouse Fc domains and hFcRn, hFcRn transgenic mice have low IgG levels^65–68^. To study the effect of competing endogenous IgG on cellular recycling, we modified the HERA protocol such that the assay could be carried out in the presence of so-called intravenous Igs (IVIg) pooled from human donors (illustrated in Fig. 7a). The protocol is described in the Supplementary Note 1, and in brief includes preloading cells with IVIg for 1 h at pH 7.4 prior to conducting HERA. As an additional modification, we performed the uptake step at pH 6.0, which enhances intracellular accumulation by allowing FcRn engagement at the cell surface and preventing receptor release during uptake^56^. Acidic pH also increases protein charge, which enhances uptake via electrostatic interaction with the negatively charged cell membrane. ABT amounts were quantified by Ag-specific ELISA, to distinguish the ABTs from the added IVIg.

We first addressed how changing the pH affected uptake and recycling. Briefly, comparing IgG1s to receptor domain-fusions demonstrated that acidic pH significantly increased the uptake of IgG1s, but not the receptor domain-fusions (Fig. 7b, c), at a magnitude of 2–3-fold. This was the case both in the absence (Fig. 7b) and presence of competition (Fig. 7c), and was mirrored by a 3–5-fold increase in recycled amounts of specifically the IgG1s (Fig. 7d, e). Thus, despite the fact that all ABMs display a pH-dependent charge, the pH modulated the cellular transport of ABTs with Fabs only. Interestingly, introducing competition did not drastically alter the hierarchy between the different ABTs in HERA (Fig. 7a–d).

Even so, the cumulative data suggested an impaired cellular recycling of the receptor domain-fusions. For the anti-TNF-α receptor domain-fusion, this was evident by its overall low amounts (Fig. 7a–d). For the anti-VEGF receptor domain-fusion, this was clear by its high relative uptake compared to the anti-VEGF IgG1 (Fig. 7a, b) not being mirrored by increased recycled amounts (Fig. 7c, d), as this signifies an increased amount of anti-VEGF receptor domain-fusion being degraded rather than recycled.

Therefore, we speculated that the receptor domain-fusions could be less potent competitors for cellular recycling than the IgG1s. To study the competitive ability of each ABT, we measured their capacity to reduce recycling of the anti-NIP IgG1. We did so by adding a constant amount of the anti-NIP IgG1 in the presence of titrated amounts of the ABTs at pH 6.0 to preloaded cells. After uptake, we added medium with pH 7.4 and incubated for 3 h to allow for cellular recycling. The amounts of anti-NIP IgG1 present in the collected medium were quantified by Ag-specific ELISA, which revealed that higher amounts of the receptor domain-fusions than IgG1s were required to reduce recycling of anti-NIP IgG1. Specifically, the IC50 value of both receptor domain-fusions were at least 2-fold that of the IgG1s with corresponding specificities (Fig. 7f, g), demonstrating a reduced capacity of receptor domain-fusions to undergo cellular recycling.

Strikingly, the Fc fragment demonstrated an IC50 of 32.9 nM, and thus reduced rescue of anti-NIP IgG1 more potently than the ABTs. Thus, the cumulative data demonstrates that the absence of Fc-fused moieties enhances not only intracellular accumulation and recycling, but also ability to compete for recycling.

## Discussion

FcRn is a key regulator of the circulatory half-life of IgG and all Fc-containing ABTs, due to its ability to bind to the Fc fragment in a pH-dependent fashion^2,21,22^. Even though the Fc harbors the principal binding site for FcRn, ABTs with identical Fc sequences, but distinct biophysical differences in their Fab arms or Fc-fusion partners, exhibit different plasma half-lives. This is a result of both FcRn-dependent and -independent factors^2,4,45,69,70^. While Fc-fusions in general have shorter plasma half-life than full-length IgG1s, little is known about their ability to engage FcRn and their cellular transport properties. Therefore, we conducted a comprehensive study of FcRn engagement and cellular transport of IgG1 Fc-based molecules.

When comparing plasma half-life of an IgG1 and its Fc fragment in hFcRn transgenic mice, we found it to be considerably shorter for Fc than for IgG1. Importantly, this does not stem from different in vitro binding kinetics to the receptor, as they bound equally well in SPR. Rather, the presence of the Fabs extends plasma half-life by a mechanism that involves FcRn expression on cells. Then, by the use of mouse strains with different hFcRn expression levels, we showed that the amount of FcRn present affects the extent of Fab-mediated half-life extension. We chose a hapten-specific chimeric mouse-human anti-NIP IgG1 as a model Ab, which had an 8.5-day plasma half-life in hemizygote Tg32 mice, in line with a previous report^56^. While the half-life of distinct IgGs varies in this model, most exhibit plasma half-lives in the range of 4–8 days^71^, which is at least 2-fold longer than the 1.8 days we measured for the Fc fragment. Interestingly, in a recent study of a human IgG4 Fc fragments engineered to lack FcRn-binding, a 5.6-fold reduction in plasma half-life in non-human primates was seen^72^. This study supports our finding that the Fc fragment (50 kDa), regardless of some renal elimination, is indeed rescued from degradation in an FcRn-dependent manner in vivo.

We found that intracellular uptake of the Fc fragment is more efficient than that of IgG1, and that the uptake is FcRn dependent. Recycling is also enhanced. However, as uptake increased more than recycling, we found the Fc fragment to accumulate within cells, and more so than IgG1. Intracellular degradation of this fraction would partly explain the short serum half-life of the Fc fragment.

Furthermore, as the Fc fragment is taken up more efficiently than IgG1, it potently outcompeted the IgG1 for cellular recycling. Last, but not least, the Fc fragment was transcytosed at an efficiency beyond that of IgG1 in polarized kidney cells. We speculate that this may be a direct effect of greater uptake of Fc on the apical side.

Recent reports highlight that pI and surface charge distribution of Abs may influence both FcRn-dependent and -independent parameters, and that high positive charge in particular may delay release from FcRn^45,48–50^. However, as we found our model IgG1 to have a more positive net charge than the Fc throughout the pH-gradient, positive charge cannot explain the increased uptake and subsequent transport of the Fc fragment. We postulate that it could rather be associated with sterical hindrance caused by Fc-fused structures upon binding to FcRn in the curved membrane of the endosomes. The Fc binds FcRn in a 1:2 stoichiometry in an orientation that brings the Fabs, and conversely, Fc-fused structures in general, into close proximity with the membrane^39,73^. Accounting for this, a two-pronged binding mechanism has been proposed, where the Fabs bend away from the membrane to interact with FcRn^51^. The ease at which Fc-fused structures can undergo such a reorientation upon binding to FcRn may affect the spatial orientation and stability of the Fc:FcRn complex in the endosome. As such, unfavorable biophysical characteristics of Fc-fused structures may impose on FcRn engagement. A sole Fc will have no such restrictions, which could explain its specific FcRn-related properties observed in our findings.

Consequently, we went on to compare the naked Fc fragment with Fc-fusions containing Ag-binding receptor domains. This revealed that neither receptor domain-fusion impair in vitro binding to FcRn, and that their presence rather increases plasma half-life 2-fold relative to that of the Fc. In comparison, in the same mouse strain, the model IgG1 had a half-life more than 6-fold longer than the Fc. This demonstrates that the presence of the Fab arms contributes to FcRn-dependent pharmacokinetics, in a manner distinct from that of Fc-fused receptor domain domains.

Notably, the anti-TNF-α receptor domain Fc-fusion etanercept displayed a near 12-fold higher concentration in plasma than both the Fc fragment and the anti-VEGF receptor domain Fc-fusion aflibercept 24 h after injection. This could be explained by high intracellular accumulation of the Fc fragment and aflibercept relative to etanercept. Both aflibercept and the Fc-fragment were internalized in an FcRn dependent manner, while etanercept chiefly stayed in solution. Interestingly, the high plasma levels of etanercept did not translate into a longer plasma half-life, as this was approximately the same as for aflibercept. Thus, distribution to predominantly plasma, rather than cells and tissues, does not necessarily prolong plasma half-life.

To learn more about how differences in the Fc-fused moiety affect FcRn-mediated processes, we included both the two receptor domain-fusions and three IgG1s targeting either VEGF or TNF-α in further studies. All had similar FcRn-binding properties at acidic pH in SPR and affinity chromatography, that is, in the absence of a membrane. Moving on to cellular studies, however, where the receptor is embedded in the membrane, we observed that while the anti-VEGF receptor domain Fc-fusion aflibercept, similar to the Fc fragment, was taken in and recycled quite efficiently, it also accumulated intracellularly. This allows for subsequent degradation. All three IgG1s were internalized and recycled efficiently. However, much less anti-VEGF IgG1 bevacizumab accumulated intracellularly. It was thus rescued efficiently, in line with it having a particularly long plasma half-life, twice that of the two other IgG1s.

Upon injection into patients, IgG1 Fc-based ABTs will compete for binding to FcRn in the presence of endogenous IgG, which has an average concentration in blood of 12 mg/ml. The fact that the half-life of IgG molecules is controlled by a concentration-catabolism relationship^74^, became evident by studies of IgG in myeloma patients and mice preloaded with IgG^75,76^. Lack of such competition prolongs half-life of IgG^67,77^, and while recent progress has been made to account for endogenous competition in transgenic mouse models^76,78^, in vivo studies of the impact of competition on different ABT designs are limited by ethical challenges, high cost and animal availability. Therefore, we established a HERA protocol where endogenous competition for the IgG-binding site of the receptor is included, which confirmed that receptor domain-fusions are less efficient than IgG1s in cellular recycling.

Notably, efficient cellular uptake of Abs correlates with high net positive charge, facilitating interaction with the negatively charged cell membrane. This is exemplified by the anti-p40 IgG1 briakinumab, which has large positive charge patches in the variable domains of its Fab arms that delay its release from FcRn at neutral pH^45^ and enhances its unspecific cellular uptake in endothelial cells^79^. This gives briakinumab a plasma half-life of only 8–9 days in humans^80,81^. In line with this, briakinumab displayed a high uptake in HERA, which was not significantly reduced upon siRNA treatment (Supplementary Fig. 1b). For our chosen ABTs, charge differences largely correlated with their cellular uptake. For example, the ABT with the highest uptake in HERA was the anti-VEGF receptor domain-fusion aflibercept, the ABM of which has a higher positive charge than any of the other Fc-fused structures. Furthermore, comparing the anti-TNF-α IgG1s revealed a higher uptake of adalimumab, which exhibits a higher Fv net charge than infliximab. Interestingly, FcRn knockdown by siRNA reduced the intracellular levels of adalimumab to a similar range as that of infliximab, suggesting that differences in cellular transport between these two IgG1s could be due to FcRn-related processes.

All findings described so far concern ABTs in the absence of their cognate Ags. When used clinically, the cognate Ag will be present and bind to the ABT in question. This masks the AIRs, adds charge intrinsic to the Abs, and also increases the avidity of the Fc:FcRn interaction after immune complex formation, thus altering two important biophysical properties; (1) overall size and (2) charge. How ABTs interact with their cognate Ags dictate their complex formation properties, which again affect their signaling properties through Fcγ receptors, biodistribution and plasma half-life^82–85^. For example, the anti-VEGF IgG1 bevacizumab, but not the anti-VEGF receptor domain Fc-fusion aflibercept form large, multimeric immune complexes with the VEGF dimer^86^. Similarly, both anti-TNF-α IgG1s form large immune complexes upon binding to Ag, while the receptor domain-Fc-fusion etanercept rather forms unstable, heterogenous 1:1 and 1:2 immune complexes with the TNF-α trimer, where the trimer may dissociate into monomers^61,87,88^. Our findings support this, as we found IgG1s, but not receptor domain-fusions to form large immune complexes in the presence of cognate Ag.

The size of immune complexes has been shown to predict recycling via FcRn in HMEC-1 cells, with larger immune complexes being sorted to lysosomal degradation rather than recycling tubules^57^. However, FcRn can also prolong the half-life of Ags by recycling and transcytosing Ag-bound IgG^31,89,90^, and FcRn blockade reduces the amounts of immune complexes in circulation^35^. Elaborating on this, we found that immune complexes of similar size displayed distinct differences in their pH-dependent binding to FcRn and cellular sorting in HERA. In general, however, Fc-multimerization increased the association and decreased the dissociation from FcRn in SPR. Furthermore, it invariably increased both intracellular uptake and recycling in HERA, but whether the increased uptake was FcRn dependent varied independently of immune complex size.

Thus, other factors must be affecting the cellular transport of Ag-bound ABTs. We found that pH modulates Ag-binding for all ABTs with a pH-dependent charge in their AIRs. Of note, our charge analyses revealed an inherent negative charge of both TNF-α and VEGF of different magnitudes. The difference in Ag charge will be amplified in solution, due to the respective trimerization and dimerization of TNF-α and VEGF, yielding a substantial negative charge of TNF-α bound ABTs compared to VEGF-bound ABTs. On the FcRn-column, we observed that these Ag-associated differences affected dissociation from the receptor for specifically TNF-α bound ABTs. As this was the case for both the large immune complexes formed by IgG1s and the smaller, less stable receptor domain-fusion complex, this must be due to charge, rather than immune complex size. VEGF-binding affected dissociation for neither anti-VEGF molecule.

In the cellular context, however, we again observed that these biochemical observations did not predict FcRn-mediated transport. Despite the fact that all anti-TNF-α ABTs were affected by Ag-binding on the column, only the IgG1s were affected in HERA. Furthermore, while Ag-binding increase the uptake of both IgG1s, the increase was FcRn-dependent for adalimumab only. In contrast, VEGF-binding drastically increased the uptake of both the IgG1 and receptor domain-fusion, but in an FcRn independent manner. However, subsequent recycling was much more efficient for the large IgG1 immune complex than the small VEGF-bound receptor domain-fusion complex, which was rather retained within cells. As the anti-VEGF IgG1 was the only ABT with negatively charged AIRs, this suggests that the combination of negative charge and Fc-multimerization had a distinct impact on cellular transport.

Interestingly, whether Ag-binding enhanced cellular accumulation of ABTs correlated with the presence of pH-dependent charge in their AIRs, as the receptor domains of the anti-TNF-α Fc-fusion etanercept was the only Fc-fused moiety without such charge. Consequently, etanercept was the only ABT to not exhibit increased uptake when bound to its Ag. This was the only noticeable charge anomaly for the receptor domains of etanercept, as they lack pH-dependent surface charge patches and possess an overall net charge similar to the Fv of the anti-TNF-α IgG1 infliximab. Thus, for this particular Fc-fusion, receptor domain-specific traits, other than large charge variations, must be the cause of reduced capacity to undergo FcRn-mediated processes. This could be due to structural flexibility of the receptor domain-domains, which could affect their orientation relative to the membrane upon binding to FcRn. Another possibility is differences in N-linked glycosylation. Both the aflibercept and the etanercept receptor domains harbor tentative N-glycosylation sites (4 and 2, respectively, predicting from the amino acid sequence N-*X*-S/T^91^), and the only such site in the IgG1s included in our studies is found in the infliximab Fv. N-glycans may affect antibody binding to both effector molecules and FcRn^92^. Whether such effects could explain the characteristic cellular transport of Fc-fusions, should be accounted for in future studies.

While Ag-binding affected the uptake and recycling in HERA differently for the various ABTs, the effect of Ag-binding on transcytosis was rather similar for all. Thus, how the distinct biophysical properties of both ABTs and Ags affect cellular transport clearly vary between transport pathways.

From a methodological perspective, the present findings reveal that studies of FcRn-mediated processes must carefully consider (1) the embedment the receptor in the cell membrane, evident by the lack of correlation between binding kinetics and cellular transport, and (2) the competitive pressure on the IgG-binding site on FcRn, evident by the impaired recycling efficiency of receptor domain-fusions yielding a poor competitive ability in HERA. We speculate that accounting for both these factors may allow for prediction of how the biophysical properties of IgG1 Fc-based ABTs dictate the nature and outcome of their interaction with FcRn.

While the Fab arms have evolved to grasp specific Ags, they may also modulate cellular handing and the ability to engage FcRn. Consequently, this results in a distribution of antibodies between the extracellular milieu and intracellular endosomal space, as well as between the blood, lymphatic system and mucosal tissue and surfaces. Importantly, complex formation and the biological fate of Ag-bound ABTs varies across molecular designs^82,86^, and others have found receptor domain-containing Fc-fusions to exhibit reduced binding to FcRn compared to IgG1s^93^. Our study supports this, and demonstrates that Fc-fused modalities have very distinct effects on immune complex formation and cellular handing. We show that these effects do not relate directly to their FcRn-binding capacity at acidic pH in the absence of their Ag, but rather to the biophysical differences of both ABTs and Ags, which affect the ability of ABTs to interact with FcRn in cellular membranes and be transported. In conclusion, our findings inform on the outcome of fusing different moieties to the IgG1 Fc. Such knowledge improves our understanding of antibody biology, as well as guide the design and preclinical testing of ABT candidates.

## Methods

### Protein acquisition

cDNA sequences encoding the HC of IgG1 Fc were designed based on its amino acid sequence as defined by the Uniprot entry IGHG1_HUMA. and synthesized in a pFuse vector with zeocin resistance by Genscript Biotech Corporation. Generation of the anti-NIP IgG1 has been previously described^52,53^. Both the IgG1 Fc and anti-NIP IgG1 were produced in human embryonic kidney (HEK) 293E cells, essentially as described^53,56^. Vectors were transiently transfected into HEK293E cells using Lipofectamine 2000 (Life Technologies) followed by harvesting and replacement of growth medium every second day for 2 weeks. Both proteins were purified on CaptureSelect (Thermo Fisher) affinity columns (anti-CH3 for IgG1 Fc and anti-CH1 for anti-NIP IgG1, respectively) prior to SEC on a Superdex 200 Increase 10/300 GL column (GE Healthcare) coupled to an Äkta FPLC instrument (GE Healthcare). Finally, monomeric fractions were upconcentrated on Amicon Ultra-15 ml columns (Millipore) with respective cut-offs at 10 K and 50 K for IgG1 Fc and anti-NIP IgG1. Purified fractions were tested by non-reduced and reduced SDS-PAGE and a two-way anti-Fc ELISA as described below. Truncated monomeric His-tagged FcRn was produced in a Baculovirus expression system as described^56,94^. Controls (briakinumab and anti-NIP IgG1s MST/HN and IHH) were produced as described^56^ (Grevys et al., manuscript in preparation). ABTs, specifically etanercept (Enbrel™), aflibercept (Eylea™), infliximab (Remicade™), adalimumab (Humira™), bevacizumab (Avastin™), eftrenonacog alfa (Alprolix™) and IVIg (Panzyga™), were acquired from the pharmacy. Etanercept, aflibercept, infliximab, adalimumab, bevacizumab and eftrenonacog alfa were buffer-exchanged into sterile phosphate-buffered saline (PBS) and run on SEC as described above prior to starting experiments.

### Cell culture

The HEK293E cell line (ATCC) was cultured in RPMI-1640 GlutaMAX-containing medium (Sigma-Aldrich) supplemented with 10% fetal calf serum (FCS) 25 U/ml penicillin (Sigma-Aldrich) and 25 μg/ml streptomycin (Sigma-Aldrich). The MDCK cell line stably expressing hFcRn (MDCK-hFcRn) was generated by Drs. Jens Fischer and Alex Haas (Roche Pharma Research and Early Development), and cultured in cultured in DMEM with 15% FCS, 25 U/ml penicillin and 25 μg/ml streptomycin, as well as 100 μg/ml G418 (Sigma-Aldrich) to maintain expression of FcRn. HMEC-1 cells stably expressing HA-FcRn-EGFP (HMEC-1-FcRn^57^) were cultured in MCDB131 medium (Gibco) supplemented with 2 mM L-glutamine, 25 μg/ml streptomycin, 25 U/ml penicillin, 10% FCS, 10 ng/ml mouse epidermal growth factor (BD Biosciences) and 1 μg/ml hydrocortisone (Sigma-Aldrich), and 100 μg/ml G418 and 50 μg/ml blasticidine (InvivoGen) to maintain FcRn expression. Percentage of HMEC-1 cells expressing HA-FcRn-EGFP in culture was above 85%, which was verified by visualizing EGFP-positive live cells, stained with the amine-reactive dye ViViD (Invitrogen), in combination with a biotinylated anti-FcRn mAb (ADM31^95^) and allophycocyanin-conjugated streptavidin (PhycoLink) using a BD LSRFortessa.

### ELISA

ELISA was performed to quantify protein levels in plasma samples, cell lysates and medium samples from cellular experiments. All quantification experiments were performed with the molecule to be analyzed as its own standard. Briefly, in a two-way anti-Fc ELISA, polyclonal anti-Fc (Sigma-Aldrich) from goat was diluted 1:5000 in PBS and coated by adding a volume of 100 μl in ELISA wells and incubating at 4 °C overnight (ON). Next, all wells were blocked by adding 250 μl of PBS supplemented with 4% skimmed milk (M) and incubating on a tabletop shaker for 1 h at room-temperature (RT). Plates were washed four times with 200 μl PBS containing 0.05% Tween20 (T; PBST) between all subsequent steps. Plasma and cellular samples were then added in 100 μl at a final dilution ranging from 1:50 to 1:400 (plasma) and 1 to 16 (cellular samples) in PBSTM. Following a 1-h incubation at RT with shaking, a polyclonal anti-human Fc from goat conjugated to alkaline phosphatase (Sigma-Aldrich) was diluted 1:5000 in PBSTM and added at a final volume of 100 μl per well. After a final 1-h incubation at RT with shaking, 100 μl of 10 μg/ml *p-*nitrophenyl-phosphate substrate (Sigma-Aldrich) dissolved in diethanolamine solution was added to all wells to develop the ELISA. A Sunrise spectrophotometer (Tecan) was used to measure absorbance at 405 nm. For capture of ABTs on cognate Ag, 500 ng/ml of cognate Ags (VEGF-165 (Sino Biologics), TNF-α (Peprotech) or NIP-conjugated bovine serum albumin (BSA-NIP25)) were coated in a final volume of 100 μl. Subsequent steps in Ag-binding ELISA were performed as described for the two-way anti-Fc ELISA.

### Animal experiments

FcRn KO mice (B6.129 × 1-Fcgrt tm1Dcr/Dcr), Tg32 hemizygous hFcRn transgenic mice (B6.Cg-Fcgrt tm1Dcr Tg(FCGRT)32Dcr/DcrJ) and Tg32 homozygous hFcRn transgenic albumin knock-out mice (B6.Cg-Albem12Mvw Fcgrttm1Dcr Tg(FCGRT)32DcR/MvwJ) received 0.8 mg/kg IgG1 Fc or 2.6 mg/kg anti-NIP IgG1. In a separate experiment, homozygous Tg32 mice also received 1.7 mg/kg aflibercept, 0.8 mg/kg IgG1 Fc,1.8 mg/kg etanercept and 1.7 mg/kg eftrenonacog alfa. All experiments were done with a mix of male and female aged 8–10 weeks, with a weight of 20–30 g/mouse and five mice per group. For experiments with IgG1 Fc and anti-NIP IgG1, 25 μl blood samples were obtained from the retro-orbital sinus vein at either 1, 1.5, 2, 2.5, 3, 3.5, 4, 5, 6 and 7 days after injection (FcRn KO mice), or 1, 1.5, 2, 2.5, 3, 3.5, 4, 5, 7, 10, 12, 16, 19, 23 and 30 days after injection (hemizygote Tg32 mice), or 1, 2, 3, 5, 7, 10, 12, 12, 16, 19, 23, 30 and 37 days after injection (homozygote Tg32 mice). In the separate experiment where homozygous Tg32 mice were dosed with IgG1 Fc, aflibercept or etanercept, blood sampling was performed as previously described at 1, 2, 3, 4, 5, 7, 10, 12, 16, 19 and 23 days after injection. All blood samples were mixed with 1 μl % K3-EDTA and kept on ice up until centrifugation at 4 °C for 5 min at 17,000 × *g*. Isolated plasma was diluted 1:10 in 50% glycerol/PBS and stored at −20 °C until analysis. All animal studies were performed at The Jackson Laboratory (JAX Service, Bar Harbor, ME), in accordance with guidelines and regulations approved by the Animal Care and Use Committee at The Jackson Laboratory. Protein plasma concentrations were quantified by the two-way anti-Fc ELISA as described above.

Plasma levels of the Fc-containing molecules were determined by nonlinear regression in GraphPad Prism (version 8.3.1, GraphPad Software LLC) using a log(agonist) vs. response equation for variable slopes. Levels at the end of the presumed the α-phase (i.e., after 24 h) were set to 100% and plasma concentrations calculated as a percentage relative to that level. A nonlinear fit of the resulting values to a semilogarithmic model was done to visualize compound elimination. β-phase half-life was calculated in Microsoft Excel version 16.16.19 (200210) by the equation *t*_1/2_ = log 0.5(log*A*_x_*/A*_0_)*t, where *A*_0_ is the amount entering the β-phase, and *A*_x_ is the amount calculated at the given time (*t*). For further pharmacokinetic parameters, the molecular concentrations determined from ELISA were fitted to a non-compartmental pharmacokinetic model using the gPKDsim add-on for MatLab^55^.

### Preincubation of ABTs with cognate Ags

For all experiments involving preincubation of ABT with cognate Ags, preincubation was done by adding the specified ratios of Ag and ABT in a volume of 20 μl of the appropriate buffers, mixing thoroughly and incubating at RT for 20 min prior to experiment initiation. Where injection of such samples is specified, the injection, preincubation and running buffers were all the same.

### Analytical size-exclusion chromatography

Analytical SEC was performed using a Superdex 75 10/300 column coupled to an Äkta FPLC instrument (Cytvia). Injections were performed in either sterile PBS (Gibco) for pH 7.4 studies or PBS adjusted to pH 5.5 with 1 M MES for cell culture (Sigma). In total, 1 μg/ml of the ABTs was injected in a total volume of 20 μl. For studies of complex formation, 1 μg/ml of ABTs were preincubated with the specified molar ratios of their cognate Ags in an end volume of 20 μl of either PBS pH 7.4 or 5.5 for 20 min at RT prior to injection.

### Analytical FcRn chromatography

Analytical FcRn affinity chromatography was performed using an Äkta Avant25 instrument (Cytvia), as described^56^. In total, 77 µl of a 1 mg/ml protein solution was injected on a column with FcRn-coupled resin in a pH 5.5 buffer (20 mM MES, 140 mM NaCl), and eluted through a lineal pH gradient obtained by gradual increase of a pH 8.8 buffer (20 mM Tris-HCl, 140 mM NaCl) throughout 110 min. Accurate pH elution values were determined by use of a pH monitor (Cytvia). For IC studies, 1 mg/ml of the ABTs were preincubated with their cognate Ags as described above, here in the 20 mM MES, 140 mM NaCl buffer, prior to dilution into the specified injection volume and column application.

### Surface plasmon resonance

SPR experiments were performed using a Biacore T200 instrument (Cytvia). Binding of ABTs to cognate Ags was studied by immobilizing ~300 resonance units (RU) of either VEGF-165 or TNF-α on CM5 sensor chips using the manufacturer’s amine coupling kit. In total, 100 nM of each ABT was diluted in a running buffer of either pH 5.5 (67 mM phosphate, 0.15 M NaCl, 0.005% Tween20) or adjusted to pH 7.4 with 1 M NaOH, and injected at a flow rate of 20 μl/min. Also, ABTs were preincubated for 20 min at RT in a volume of 20 μl with 200 nM monomeric, His-tagged FcRn in the pH 5.5 running buffer, followed by injection over the respective cognate Ags. Ten mM NaOH at 20 μl/min was used for regeneration. All individual injections were normalized to baseline, all sensorgrams zero-adjusted in the BIAevaluation software version 4.1 (GE Healthcare), and all shown data have been subjected to blank subtraction of the response on a flow cell from the same chip.

Binding kinetics of the monomeric ABTs to FcRn were determined by immobilizing the ABTs at ~400 RU on CM5 chips with the manufacturer’s amine coupling kit. Serial dilutions of monomeric His-tagged FcRn (2000-1.95 nM) were prepared and injected in the pH 5.5 running buffer at 50 μl/min. Regeneration was done using pH 7.4 HBS-P + (0.15 M NaCl, 0.1 M HEPES, 0.005% surfactant P20) at 30 μl/min with contact and stabilization periods of 30 s.

Binding of Ag-bound ABTs to FcRn was determined by immobilizing site-specifically biotinylated FcRn (Immunitrack) on SA-chips at ~100 RU. ABTs (15.62 nM) were injected either alone or following preincubation with cognate Ags as previously specified in the pH 5.5 running buffer at a flowrate of 50 μl/min. Regeneration was done with HBS-P + pH 7.4.

### HERA

For HERA experiments, 3.75 × 10^4^ HMEC-1-FcRn cells were seeded in a volume of 250 μl in Costar 48-well plates in the culturing medium 20–24 h prior to experiment initiation. Three wells were seeded per experimental parameter. Following visual inspection to confirm characteristic morphology and sufficient confluency, the medium was removed from all wells and the cells washed thrice in 250 μl RT Hank’s balanced salt solution (HBSS, Gibco). After washing, the cells were starved for 1 h in 250 μl RT HBSS.

For studies of the ABTs in the presence of cognate Ag, ABTs were next prepared at a final concentration of 800 nM in RT HBSS either alone or following preincubation in 25 μl for 20 min at RT with two-fold molar excess of their cognate Ags, then added to cells at a final volume of 125 μl in technical triplicates. For studies of anti-NIP IgG1 and the Fc fragment, both molecules were prepared as 800 nM samples and added to cells as specified for ABTs. After a 3-h incubation, the samples were removed and the cells washed three times in 250 μl ice-cold HBSS. At this time, uptake plates were frozen at −80 °C following aspiration of washing medium, while recycling plates received 220 μl RT serum-free growth medium supplemented with MEM non-essential amino acids. After another 3-h incubation, recycling samples were harvested and washing with ice-cold HBSS repeated. Resulting residual plates were frozen and stored at −80 °C to the day of analysis.

For competition studies, cells were incubated for 1 h in 125 μl HBSS supplemented with 0.5% IgG-depleted FCS (Gibco) either with or without 7.5 mg/ml (unless elsewise specified) IVIg (Octapharma), depending on the specified competition setting (i.e., preloading of cells). Following preloading, cells were washed twice in 250 μl ice-cold HBSS, pH 7.4, before adding samples at a concentration of 400 nM in a final volume of 125 μl. Where specified, samples were prepared in HBSS adjusted to pH 6.0 by adding 7 mM MES buffer (Sigma-Aldrich). For studies of the capacity of Fc-containing molecules to reduce recycling of anti-NIP IgG1, this step was performed with a titration series with final concentrations 6400-1 nM of each ABT were prepared with 400 nM anti-NIP IgG1 in pH 6.0 HBSS. Storage of cell plates, incubation, washing, harvesting of recycling samples was done as described above.

At the day of analysis, recycling samples were thawed at RT, and frozen cells were lysed by adding 220 μl RIPA buffer (Thermo Fisher) supplemented with cOmplete protease inhibitor cocktail (Roche) and incubating on ice on a shaker for 10 min. Cellular debris was removed by 5 min centrifugation at 10,000 × *g* and proteins quantified by ELISA as described above. Samples were analyzed by ELISA as described above. Specifically, samples for quantification of the IgG1 Fc fragment and ABTs preincubated with cognate Ags were analyzed by the two-way anti-Fc ELISA. Samples from experiments involving preloading cells with IVIg were analyzed by Ag-binding ELISAs, with the exception of quantification of total amount of IgG in cells, which was done by the two-way anti-Fc ELISA.

### siRNA knockdown of FcRn expression in HERA

HERA in siRNA-treated cells was performed as described^56^. Briefly, 3.75 × 10^4^ HMEC-1-FcRn cells were seeded in a total volume of 500 μl in 24-well Costar plates in the culturing medium 20–24 h prior to siRNA transfection. Transfection with mixtures of FcRn-HC-specific siRNA (sc-45643, Santa Cruz Biotechnology Inc) and control siRNAs (sc-37007) was performed as described by the manufacturer. After transfection, cells were incubated for 48 h before performing the uptake phase of HERA as described above, with 400 nM end concentration of the specified analytes in a volume of 250 μl being added to cells followed by an incubation of either 0.5 or 2 h.

### Transcytosis studies

In total, 1.12 cm^2^ diameter transwell filters with a 400 nm pore size and collagen-coated polytetrafluoroethylene membranes (Corning Costar) were incubated ON in the cell incubator with complete growth medium prior to seeding of 1–1.25 × 10^6^ MDCK-FcRn cells/well. Cells were incubated for 18–24 h to reach confluence and a transepithelial electrical resistance of 7–800 Ω cm^2^. Next, both chambers were washed two times with 500 μl RT HBSS prior to starving cells for 1 h in 500 μl RT HBSS. Next, 200 nM analyte was added to the top filter in an end volume of 200 μl. Cells were then incubated for 4 h in the cell incubator before harvesting samples from the bottom chamber. Protein levels were determined by the two-way anti-Fc ELISA as described above. For ABT:Ag studies, ABTs were preincubated with their cognate Ags as described above before being diluted to an end concentration of 200 nM and added to cells.

### Protein sequence alignment and sequence numbering

Amino acid sequences were aligned using ClustalOmega and Jalview 2.10.5. Where specified, amino acid residue positions refer to standard EU numbering as defined by IGHG1_HUMA, Uniprot accession #P01857.

### Visualization of crystal structures and surface charge

Structural and surface charge analyses were done based on solved X-ray crystallographic structures retrieved from the PDB of bevacizumab (PDB ID: 1BJ1), adalimumab (PDB ID: 3WD5), infliximab (PDB ID: 4G3Y), and etanercept (PDB ID: 3ALQ). For aflibercept, a homology model was generated by using the structures of VEGFR1 domain 2 (PDB ID: 1QTY) and VEGFR2 domain 3 (PDB ID: 3V2A). The connecting linker and missing loops in aflibercept were modeled using Rosetta Remodel^96^. Ag structures were isolated from 3ALQ (chain A + B + C) and 1BJ1 (chain V + W).

To visualize surface charge, pqr files were generated using the pdb2pqr web server version^97^ with default settings. Electrostatic potentials at pH 5.5 and 7.4 were calculated as indicated, by use of PARSE force field and APBS version 3.0^98^. The isopotential surfaces were visualized at ±3 kT/e (ABMs) or ±5kT/e (Ags) using pymol version 2.4.0. AIRs were determined as CDR loops by Chothia for Fvs and as residues with at least one atom within a 5 Å radius of an Ag atom using pymol for receptor domains.

### Calculation of net protein charge

Sequence-dependent net charge at different pH values were calculated using the Emboss iep calculator (http://www.bioinformatics.nl/cgi-bin/emboss/iep?_pref_hide_optional=0). All cysteines were assumed to form disulfide bridges. Whole ABMs were defined as the combined HC and LC sequence for Fvs and as the whole receptor domain sequence for Fc-fusions, and were assumed to have one N-terminal residue per polypeptide chain. Remaining residues were defined as the whole ABMs after removing AIRs, and were assumed to have one N-terminal residue. AIRs were not assumed to have any terminal residues.

### Statistics and reproducibility

The data is presented as mean ± SD. Unpaired Student’s *t* test was used to compare pairs of experimental parallels where specified. *t*-Tests were performed using Prism 8 (GraphPad, San Diego, CA). Two-tailed *p* values ≤0.05 were considered as statistically significant. Sample sizes were pre-determined in line with previous work with related methods, and are in all cases stated in figure Legends. For cellular experiments where statistics were applied, the sample size was generally three biological replicates, with three technical triplicates within each experiment. For animal experiments, sample size was set to 5 individual animals.

### Reporting summary

Further information on research design is available in the Nature Research Reporting Summary linked to this article.

## Supplementary information

Supplementary Information

Description of Additional Supplementary Files

Supplementary Data

Reporting Summary

## Data Availability

Raw data underlying data shown in main figures is found in the Supplementary Data, and are available from the corresponding author upon reasonable request.

## Code availability

The following software and repositories were used for data collection: Microsoft Excel version 16.16.19 (200210), GraphPad Prism (version 8.3.1, GraphPad Software LLC), BIAevaluation software version 4.1, MatLab R2021a, PyMOL version 2.4.2, Emboss iep calculator (https://www.bioinformatics.nl/cgi-bin/emboss/iep?_pref_hide_optional=0), www.drugbank.com, Clustal Omega version 1.2.4, Rosetta Remodel (doi: 10.1371/journal.pone.0024109), PDB2PQR (doi: 10.1093/nar/gkh381), The following software was used for data analysis and graphical presentations: Microsoft Excel version 16.16.19 (200210), GraphPad Prism (version 8.3.1, GraphPad Software LLC), BIAevaluation software version 4.1, MatLab R2021a, PyMOL version 2.4.2, Jalview 2.10.5, www.biorender.com. Code for generation of the aflibercept model by the use of Rosetta is available in a Github repository, as described in the accompanying Code and Software Submission Checklist, and may be accessed at the following link upon reasonable request: https://github.com/rahelf/aflibercept_model.git.

## References

[1] Lu R-M (2020). Development of therapeutic antibodies for the treatment of diseases. J. Biomed. Sci..

[2] Carter PJ, Lazar GA (2017). Next generation antibody drugs: pursuit of the “high-hanging fruit.”. Nat. Rev. Drug Discov..

[3] Duivelshof BL (2021). Therapeutic Fc‐fusion proteins: current analytical strategies. J. Sep. Sci..

[4] Rath T (2013). Fc-fusion proteins and FcRn: structural insights for longer-lasting and more effective therapeutics. Crit. Rev. Biotechnol..

[5] Lagassé HAD, Hengel H, Golding B, Sauna ZE (2019). Fc-fusion drugs have FcγR/C1q binding and signaling properties that may affect their immunogenicity. AAPS J..

[6] Network DRCR (2015). Aflibercept, bevacizumab, or ranibizumab for diabetic macular edema. N. Engl. J. Med..

[7] Papadopoulos N (2012). Binding and neutralization of vascular endothelial growth factor (VEGF) and related ligands by VEGF Trap, ranibizumab and bevacizumab. Angiogenesis.

[8] Mitoma H, Horiuchi T, Tsukamoto H, Ueda N (2018). Molecular mechanisms of action of anti-TNF-α agents—comparison among therapeutic TNF-α antagonists. Cytokine.

[9] Vaisman-Mentesh A (2019). Molecular landscape of anti-drug antibodies reveals the mechanism of the immune response following treatment with TNFα antagonists. Front. Immunol..

[10] Garcês S, Demengeot J (2017). The immunogenicity of biologic therapies. Curr. Probl. Dermatol..

[11] Fleischmann R (2014). Infliximab efficacy in rheumatoid arthritis after an inadequate response to etanercept or adalimumab: results of a target-driven active switch study. Curr. Med. Res. Opin..

[12] Schaeverbeke T (2016). Immunogenicity of biologic agents in rheumatoid arthritis patients: lessons for clinical practice. Rheumatology.

[13] Ehlken C (2014). Switch of anti-VEGF agents is an option for nonresponders in the treatment of AMD. Eye.

[14] Empeslidis T (2019). How successful is switching from bevacizumab or ranibizumab to aflibercept in age-related macular degeneration? A systematic overview. Adv. Ther..

[15] Coleman CI (2012). Dosing frequency and medication adherence in chronic disease. J. Manag. Care Pharm..

[16] Kruk ME, Schwalbe N (2006). The relation between intermittent dosing and adherence: preliminary insights. Clin. Ther..

[17] Keizer RJ, Huitema ADR, Schellens JHM, Beijnen JH (2010). Clinical pharmacokinetics of therapeutic monoclonal antibodies. Clin. Pharmacokinet..

[18] Combe B (2008). Update on the use of etanercept across a spectrum of rheumatoid disorders. Biologics Targets Ther..

[19] Shapiro AD (2012). Recombinant factor IX-Fc fusion protein (rFIXFc) demonstrates safety and prolonged activity in a phase 1/2a study in hemophilia B patients. Blood.

[20] Ipema HJ, Jung MY, Lodolce AE (2009). New drug approvals: romiplostim management of immune thrombocytopenic purpura. Ann. Pharmacother..

[21] Roopenian DC, Akilesh S (2007). FcRn: the neonatal Fc receptor comes of age. Nat. Rev. Immunol..

[22] Pyzik M (2019). The neonatal Fc receptor (FcRn): a misnomer?. Front. Immunol..

[23] Zhu X (2001). MHC class I-related neonatal Fc receptor for IgG is functionally expressed in monocytes, intestinal macrophages, and dendritic cells. J. Immunol..

[24] Brambell FWR (1966). The transmission of immunity from mother to young and the catabolism of immunoglobulins. Lancet.

[25] Akilesh S, Christianson GJ, Roopenian DC, Shaw AS (2007). Neonatal FcR expression in bone marrow-derived cells functions to protect serum IgG from catabolism. J. Immunol..

[26] Challa DK (2019). Neonatal Fc receptor expression in macrophages is indispensable for IgG homeostasis. MAbs.

[27] Montoyo HP (2009). Conditional deletion of the MHC class I-related receptor FcRn reveals the sites of IgG homeostasis in mice. Proc. Natl Acad. Sci. USA.

[28] Ward ES (2005). From sorting endosomes to exocytosis: association of Rab4 and Rab11 GTPases with the Fc receptor, FcRn, during recycling. Mol. Biol. Cell.

[29] Ober RJ, Martinez C, Lai X, Zhou J, Ward ES (2004). Exocytosis of IgG as mediated by the receptor, FcRn: an analysis at the single-molecule level. Proc. Natl Acad. Sci. USA.

[30] Spiekermann GM (2002). Receptor-mediated immunoglobulin G transport across mucosal barriers in adult life functional expression of FcRn in the mammalian lung. J. Exp. Med..

[31] Ober RJ, Martinez C, Vaccaro C, Zhou J, Ward ES (2004). Visualizing the site and dynamics of IgG salvage by the MHC class I-related receptor, FcRn. J. Immunol..

[32] Yoshida M (2004). Human neonatal Fc receptor mediates transport of IgG into luminal secretions for delivery of antigens to mucosal dendritic cells. Immunity.

[33] Yoshida M (2006). IgG transport across mucosal barriers by neonatal Fc receptor for IgG and mucosal immunity. Springer Semin Immunopathol..

[34] Qiao S-W (2008). Dependence of antibody-mediated presentation of antigen on FcRn. Proc. Natl Acad. Sci. USA.

[35] Blumberg, L. et al. Blocking FcRn in humans reduces circulating IgG levels and inhibits IgG immune complex–mediated immune responses. *Sci. Adv.* eaax9586 10.1126/sciadv.aax9586 (2019). 10.1126/sciadv.aax9586 PMC6920022 31897428

[36] Baker K, Rath T, Pyzik M, Blumberg RS (2014). The role of FcRn in antigen presentation. Front. Immunol..

[37] Baker K (2011). Neonatal Fc receptor for IgG (FcRn) regulates cross-presentation of IgG immune complexes by CD8−CD11b+ dendritic cells. Proc. Natl Acad. Sci. USA.

[38] Hubbard, J. J. et al. FcRn is a CD32a coreceptor that determines susceptibility to IgG immune complex-driven autoimmunity. *J. Exp. Med.* **217**, e20200359 (2020). 10.1084/jem.20200359 PMC7537387 32658257

[39] Burmeister WP, Huber AH, Bjorkman PJ (1994). Crystal structure of the complex of rat neonatal Fc receptor with Fc. Nature.

[40] West AP, Bjorkman PJ (2000). Crystal structure and immunoglobulin G binding properties of the human major histocompatibility complex-related Fc receptor†,‡. Biochemistry.

[41] Oganesyan V (2014). Structural insights into neonatal Fc receptor-based recycling mechanisms. J. Biol. Chem..

[42] Wang W (2011). Monoclonal antibodies with identical Fc sequences can bind to FcRn differentially with pharmacokinetic consequences. Drug Metab. Dispos..

[43] Jensen P (2015). Investigating the interaction between the neonatal Fc receptor and monoclonal antibody variants by hydrogen/deuterium exchange mass spectrometry. Mol. Cell Proteom..

[44] Neuber T (2014). Characterization and screening of IgG binding to the neonatal Fc receptor. MAbs.

[45] Schoch A (2015). Charge-mediated influence of the antibody variable domain on FcRn-dependent pharmacokinetics. Proc. Natl Acad. Sci. USA.

[46] Sun Y, Estevez A, Schlothauer T, Wecksler AT (2020). Antigen physiochemical properties allosterically effect the IgG Fc-region and Fc neonatal receptor affinity. MAbs.

[47] Rabia LA, Zhang Y, Ludwig SD, Julian MC, Tessier PM (2019). Net charge of antibody complementarity-determining regions is a key predictor of specificity. Protein Eng. Des. Sel..

[48] Boswell CA (2010). Effects of charge on antibody tissue distribution and pharmacokinetics. Bioconjugate Chem..

[49] Li B (2014). Framework selection can influence pharmacokinetics of a humanized therapeutic antibody through differences in molecule charge. MAbs.

[50] Datta-Mannan A (2015). Balancing charge in the complementarity-determining regions of humanized mAbs without affecting pI reduces non-specific binding and improves the pharmacokinetics. MAbs.

[51] Jensen P (2017). A two-pronged binding mechanism of IgG to the neonatal Fc receptor controls complex stability and IgG serum half-life. Mol. Cell. Proteom..

[52] Norderhaug L, Olafsen T, Michaelsen TE, Sandlie I (1997). Versatile vectors for transient and stable expression of recombinant antibody molecules in mammalian cells. J. Immunol. Methods.

[53] Grevys A (2015). Fc engineering of human IgG1 for altered binding to the neonatal Fc receptor affects Fc effector functions. J. Immunol..

[54] Schlothauer T (2013). Analytical FcRn affinity chromatography for functional characterization of monoclonal antibodies. MAbs.

[55] Hosseini I (2018). gPKPDSim: a SimBiology®-based GUI application for PKPD modeling in drug development. J. Pharmacokinet. Phar..

[56] Grevys A (2018). A human endothelial cell-based recycling assay for screening of FcRn targeted molecules. Nat. Commun..

[57] Weflen AW (2013). Multivalent immune complexes divert FcRn to lysosomes by exclusion from recycling sorting tubules. Mol. Biol. Cell.

[58] Vaccaro C, Zhou J, Ober RJ, Ward SE (2005). Engineering the Fc region of immunoglobulin G to modulate in vivo antibody levels. Nat. Biotechnol..

[59] Stewart MW (2012). Aflibercept (VEGF Trap-eye): the newest anti-VEGF drug. Br. J. Ophthalmol..

[60] Lu J-F (2008). Clinical pharmacokinetics of bevacizumab in patients with solid tumors. Cancer Chemoth Pharm..

[61] Schie KAvan (2016). Therapeutic TNF inhibitors can differentially stabilize trimeric TNF by inhibiting monomer exchange. Sci. Rep..

[62] Varongchayakul N, Huttner D, Grinstaff MW, Meller A (2018). Sensing native protein solution structures using a solid-state nanopore: unraveling the states of VEGF. Sci. Rep..

[63] Friedl J (2002). Induction of permeability across endothelial cell monolayers by tumor necrosis factor (TNF) occurs via a tissue factor-dependent mechanism: relationship between the procoagulant and permeability effects of TNF. Blood.

[64] Deissler HL, Sommer K, Lang GK, Lang GE (2020). Transport and fate of aflibercept in VEGF-A165-challenged retinal endothelial cells. Exp. Eye Res..

[65] Roopenian DC (2003). The MHC class I-like IgG receptor controls perinatal IgG transport, IgG homeostasis, and fate of IgG-Fc-coupled drugs. J. Immunol..

[66] Andersen J, Daba M, Berntzen G, Michaelsen TE, Sandlie I (2010). Cross-species binding analyses of mouse and human neonatal Fc receptor show dramatic differences in immunoglobulin G and albumin binding. J. Biol. Chem..

[67] Petkova SB (2006). Enhanced half-life of genetically engineered human IgG1 antibodies in a humanized FcRn mouse model: potential application in humorally mediated autoimmune disease. Int Immunol..

[68] Huggins MA, Jameson SC, Hamilton SE (2019). Embracing microbial exposure in mouse research. J. Leukoc. Biol..

[69] Spiess C, Zhai Q, Carter PJ (2015). Alternative molecular formats and therapeutic applications for bispecific antibodies. Mol. Immunol..

[70] Czajkowsky DM, Hu J, Shao Z, Pleass RJ (2012). Fc‐fusion proteins: new developments and future perspectives. EMBO Mol. Med..

[71] Tam SH, McCarthy SG, Brosnan K, Goldberg KM, Scallon BJ (2013). Correlations between pharmacokinetics of IgG antibodies in primates vs. FcRn-transgenic mice reveal a rodent model with predictive capabilities. MAbs.

[72] Lawrence SA (2021). Influence of FcRn binding properties on the gastrointestinal absorption and exposure profile of Fc molecules. Bioorg. Med. Chem..

[73] Martin WL, West AP, Gan L, Bjorkman PJ (2001). Crystal structure at 2.8 Å of an FcRn/heterodimeric Fc complex. Mol. Cell.

[74] Brambell FWR, Hemmings WA, Morris IG (1964). A theoretical model of γ-globulin catabolism. Nature.

[75] Morell A, Terry WD, Waldmann TA (1970). Metabolic properties of IgG subclasses in man. J. Clin. Invest..

[76] Low BE, Christianson GJ, Lowell E, Qin W, Wiles MV (2020). Functional humanization of immunoglobulin heavy constant gamma 1 Fc domain human FCGRT transgenic mice. MAbs.

[77] Stapleton NM (2011). Competition for FcRn-mediated transport gives rise to short half-life of human IgG3 and offers therapeutic potential. Nat. Commun..

[78] Lee C-H (2019). An engineered human Fc domain that behaves like a pH-toggle switch for ultra-long circulation persistence. Nat. Commun..

[79] Grevys, A. et al. Antibody variable sequences have a pronounced effect on cellular transport and plasma half-life. *iScience* 103746 10.1016/j.isci.2022.103746 (2022). 10.1016/j.isci.2022.103746 PMC8800109 35118359

[80] Gandhi M, Alwawi E, Gordon KB (2010). Anti-p40 antibodies ustekinumab and briakinumab: blockade of interleukin-12 and interleukin-23 in the treatment of psoriasis. Semin. Cutan. Med. Surg..

[81] Lima XT, Abuabara K, Kimball AB, Lima HC (2009). Briakinumab. Expert Opin. Biol. Ther..

[82] Rudge JS (2007). VEGF trap complex formation measures production rates of VEGF, providing a biomarker for predicting efficacious angiogenic blockade. Proc. Natl Acad. Sci. USA.

[83] Meyer T (2009). Bevacizumab immune complexes activate platelets and induce thrombosis in FCGR2A transgenic mice. J. Thromb. Haemost..

[84] Nomura Y, Kaneko M, Miyata K, Yatomi Y, Yanagi Y (2015). Bevacizumab and aflibercept activate platelets via FcγRIIa. Invest. Ophthalmol. Vis. Sci..

[85] Bournazos S, Wang TT, Dahan R, Maamary J, Ravetch JV (2017). Signaling by antibodies: recent progress. Annu Rev. Immunol..

[86] MacDonald DA (2016). Aflibercept exhibits VEGF binding stoichiometry distinct from bevacizumab and does not support formation of immune-like complexes. Angiogenesis.

[87] Scallon B (2002). Binding and functional comparisons of two types of tumor necrosis factor antagonists. J. Pharm. Exp. Ther..

[88] Kohno T, Tam L-TT, Stevens SR, Louie JS (2007). Binding characteristics of tumor necrosis factor receptor-Fc fusion proteins vs anti-tumor necrosis factor mAbs. J. Invest Dermatol. Symp. Proc..

[89] Devanaboyina S (2013). The effect of pH dependence of antibody-antigen interactions on subcellular trafficking dynamics. MAbs.

[90] Ward, E. S. & Ober, R. J. *Advances in Immunology*, Vol. 103 (2009), Elsevier. Inc. 10.1016/S0065-2776(09)03004-1 PMC4485553 19755184

[91] Pitti T (2019). N-GlyDE: a two-stage N-linked glycosylation site prediction incorporating gapped dipeptides and pattern-based encoding. Sci. Rep..

[92] Wada R, Matsui M, Kawasaki N (2018). Influence of N-glycosylation on effector functions and thermal stability of glycoengineered IgG1 monoclonal antibody with homogeneous glycoforms. MAbs.

[93] Suzuki T (2010). Importance of neonatal FcR in regulating the serum half-life of therapeutic proteins containing the Fc domain of human IgG1: a comparative study of the affinity of monoclonal antibodies and Fc-fusion proteins to human neonatal FcR. J. Immunol..

[94] Popov S (1996). The stoichiometry and affinity of the interaction of murine Fc fragments with the MHC class I-related receptor, FcRn. Mol. Immunol..

[95] Christianson GJ (2014). Monoclonal antibodies directed against human FcRn and their applications. MAbs.

[96] Huang P-S (2011). RosettaRemodel: a generalized framework for flexible backbone protein design. PLoS ONE.

[97] Dolinsky TJ, Nielsen JE, McCammon JA, Baker NA (2004). PDB2PQR: an automated pipeline for the setup of Poisson–Boltzmann electrostatics calculations. Nucleic Acids Res..

[98] Baker NA, Sept D, Joseph S, Holst MJ, McCammon JA (2001). Electrostatics of nanosystems: application to microtubules and the ribosome. Proc. Natl Acad. Sci. USA.

