# Supplementary material for: Biophysical differences in IgG1 Fc-based therapeutics relate to their cellular handling, interaction with FcRn and plasma half-life
Source: Commun Biol. 2022 Aug 18;5:832. doi: 10.1038/s42003-022-03787-x (PMC9388496; doi:10.1038/s42003-022-03787-x)
Supplement: Supplementary file 1 — Supplementary Information [file 42003_2022_3787_MOESM1_ESM.pdf]

Supplementary information

Supplementary table 1. PK parameters of model antibody and IgG1 Fc in different animal models as calculated in gPKDsim[55].

Group	Route	AUC (µg*d/mL)	Cmax (µg/mL)	CL (mL/d/kg)	MRT (d)	Vss (mL/kg)	$T_{1/2}$ (d)	R^2
Fc<br>Tg32 homozygous	IV<br>Bolus	11.8	3.5	68.9	1.9	129.6	1.8 ± 0.4	0.993
IgG1<br>Tg32 homozygous	IV<br>Bolus	442.4	30.4	4.8	21.6	102.9	16.2 ± 4.1	0.991
Fc<br>Tg32 hemizygous	IV<br>Bolus	11.1	2.6	73.8	3.0	219.1	2.4 ± 0.2	0.991
IgG1<br>Tg32 hemizygous	IV<br>Bolus	539.2	49.1	4.1	16.6	66.6	11.8 ± 2.3	0.990
Fc<br>FcRn KO	IV<br>Bolus	5.6	1.7	149.8	0.8	121.9	1.1 ± 0.2	0.991
IgG1<br>FcRn KO	IV<br>Bolus	62.1	18.4	41.5	1.4	59.9	1.4 ± 0.4	0.987

Legend:
- AUC (µg*d/mL) = Area under the curve from the first to the last measured time point.
- Cmax (µg/mL) = Maximum concentration after administration.
- CL (mL/d/kg) = Clearance.
- MRT (d) = Mean residency time (the average time the drug remains in the compartment).
- Vss (mL/kg) = Volume of distribution at steady state.
- $T_{1/2}$ (d) = Terminal half-life.
- R^2 = How well the regression for half-life determination approximates the real data points.

Supplementary table 2. PK parameters of IgG1 Fc and Fc-fusions in Tg32 homozygous mice.

Group	Route	AUC (µg*d/mL)	Cmax (µg/mL)	CL (mL/d/kg)	MRT (d)	Vss (mL/kg)	$T_{1/2}$ (d)	R^2
Fc	IV<br>Bolus	8.0	2.4	105.5	1.8	188.8	1.4 ± 0.3	0.984
Aflibercept	IV<br>Bolus	73.0	9.9	24.3	5.8	137.2	3.3 ± 0.8	0.991
Etanercept	IV<br>Bolus	28.4	64.4	7.0	6.1	42.4	5.5 ± 2.6	0.947

Supplementary Note 1

To introduce competition for cellular recycling in the HERA protocol, we sought to mirror the situation of high levels of IgG both intra- and extracellularly. To achieve competitive pressure, the intracellular levels of IgG should ideally be as high as possible. By adding increasing amounts of IVIg to cells and varying incubation times, we found that adding IVIg at pH 7.4 followed by a 1-hour incubation (i.e. preloading cells) led to a stable plateau of intracellular IgG (supplementary figure 1g), as measured in cell lysates by the two-way Fc-based ELISA. Conducting HERA on preloaded cells revealed that this plateau was reached at 7.5 mg/mL IVIg, as increasing the IVIg concentration beyond this did not lead to higher intracellular amounts at the end of the uptake step (supplementary figure 1f). Importantly, preloading with more than 5 mg/mL reduced recycling of anti-NIP IgG1 (supplementary figure 1e), as measured by Ag-specific capture followed by Fc-detection in ELISA, and this drop did not increase at IVIg amounts beyond 7.5 mg/mL. Thus, competition studies were performed by preloading cells with 7.5 mg/mL IVIg for 1 hour prior to adding the ABTs.

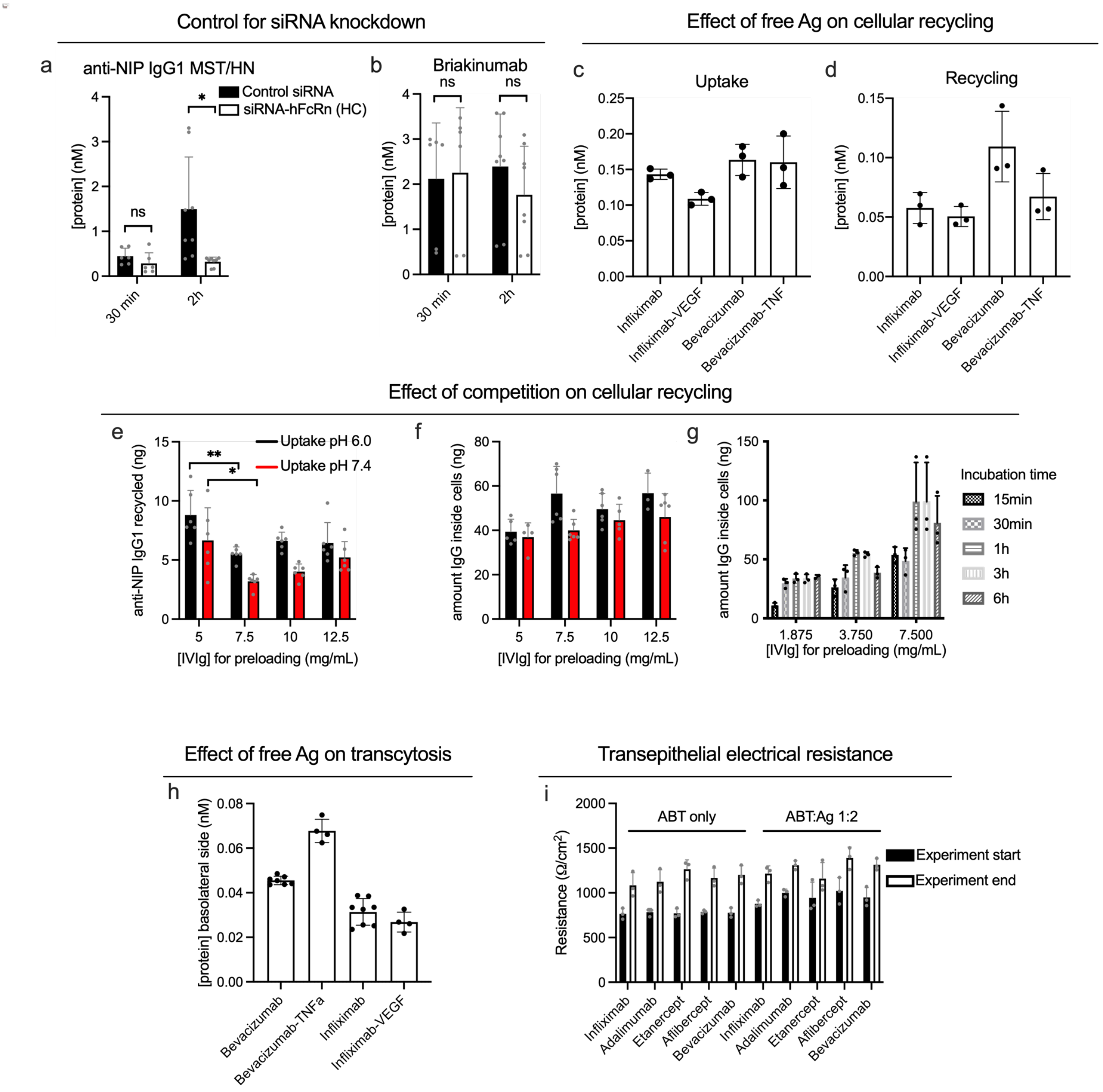

Supplementary figure 1: Additional cellular data. (a-b) Relevant controls for successful FcRn knockdown in HMEC-1-FcRn cells transfected with siRNA directed against the HC of FcRn. Values correspond to two independent experiments (n=4 per bar). (c-d) Amount of infliximab and bevacizumab (c) taken up and (d) recycled in HERA following adding of infliximab together with VEGF and bevacizumab with TNF-a. Values correspond to one experiment (n=3). (e) Amount of anti-NIP IgG1 recycled following preload with increasing concentrations of IVIg and an uptake phase at either pH 6.0 or 7.4. (f) Total amount of IgG in cells at the end of the uptake phase following preloading with increasing concentrations of IVIg. (g) Amount of IgG in cells after incubation with increasing concentrations and different incubation times. Values in (e-g) correspond to one representative experiment per graph (n=3 per bar). (h) Amount of infliximab and bevacizumab detected in basolateral chamber following MDCK-hFcRn-transcytosis of each IgG1 incubated on cells either alone or with VEGF (for infliximab) or TNF-a (for bevacizumab). Values correspond to one experiment (n=4 per bar). (i) Transepithelial electrical resistance measurements at the start and end of an experiment in the MDCK-hFcRn transcytosis assay following incubation with ABTs either as monomers or after preincubation with 2-fold molar excess of cognate Ags. Values correspond to one experiment (n=4 per bar). *$p<0.05$, **$p<0.005$ (two-tailed, unpaired Student's t-test). Data shows mean values ±SD.

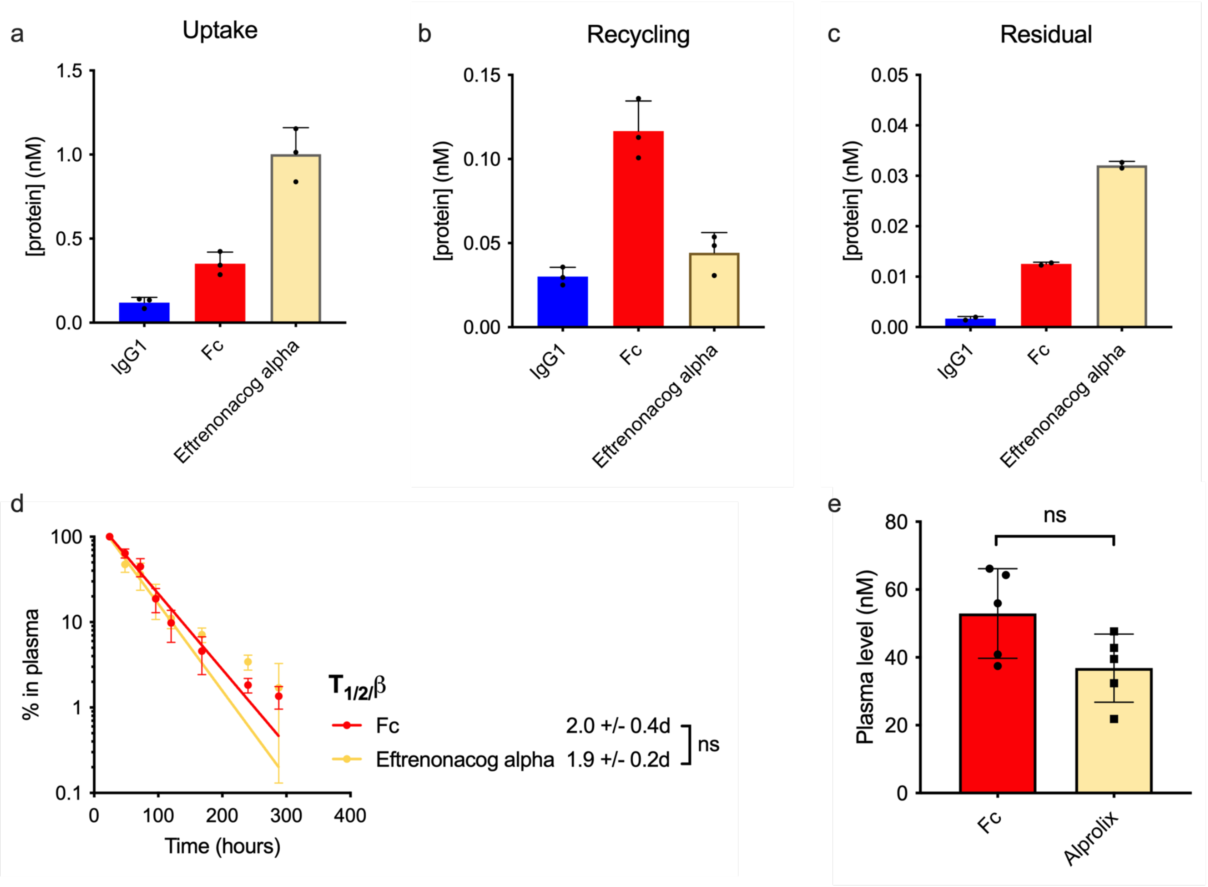

Supplementary figure 2: HERA and circulatory properties of the IgG1 Fc fragment compared to the Fc fusion efrenonacog alpha. (a-c) HERA parameters of anti-NIP IgG1 (MW≈150 kDa), (IgG1 Fc (MW≈50 kDa) and eftrenonacog alfa (MW≈98 kDa). Shown data represents one representative experiment. (d) Elimination curves and estimated β-phase half-life of IgG1 Fc and eftrenonacog alfa in homozygous Tg32 mice. (e) Molar amounts in plasma at the start of the β-phase (1 day after IV injection) in the same mice as shown in (d). Data shows mean values ±SD.

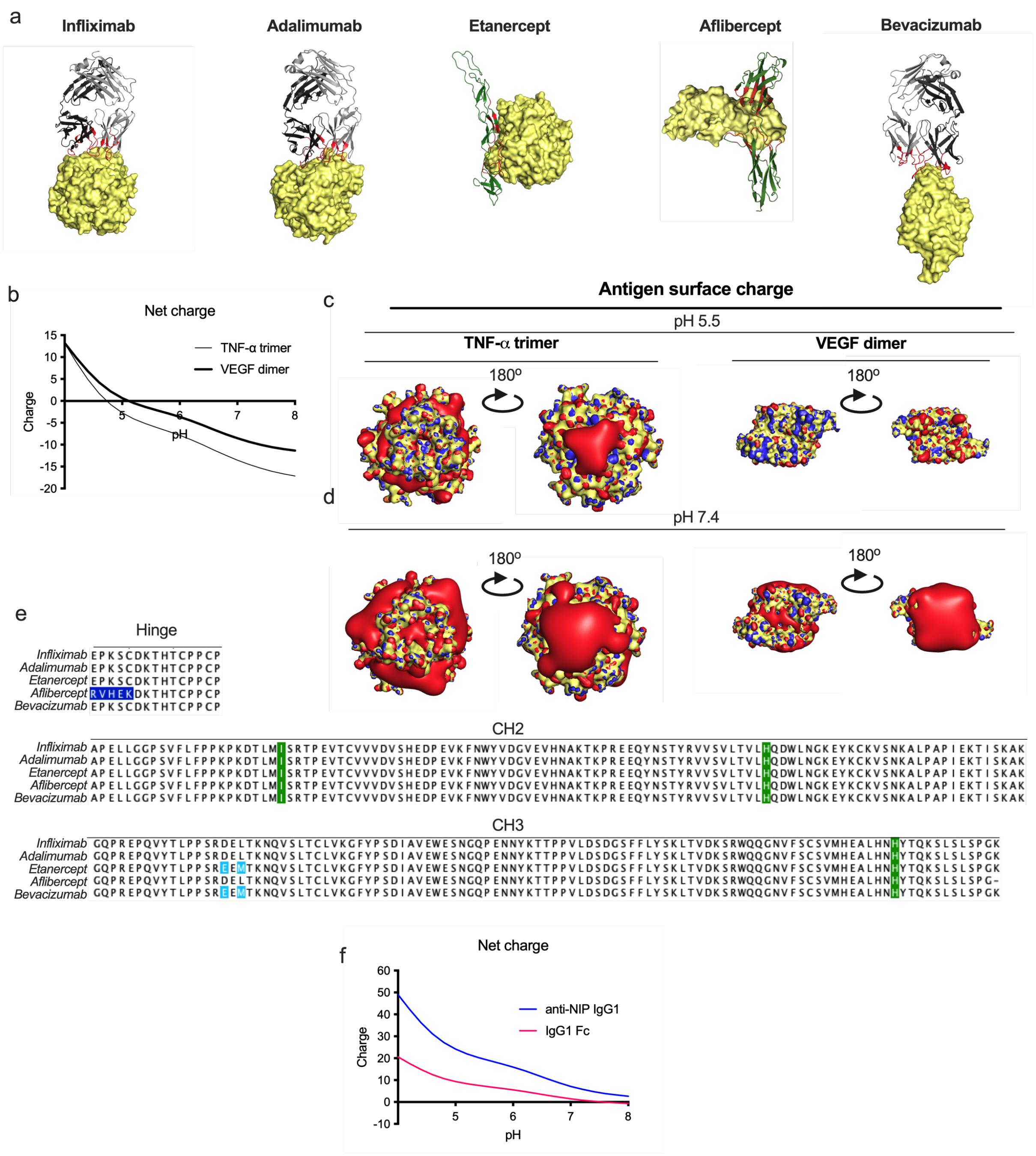
a
Infliximab
Adalimumab
Etanercept
Aflibercept
Bevacizumab
b
Net charge
TNF-α trimer
VEGF dimer
Charge
pH
15
10
5
0
-5
-10
-15
-20
5
6
7
8
c
Antigen surface charge
pH 5.5
TNF-α trimer
VEGF dimer
180º
180º
d
pH 7.4
180º
180º
e
Hinge
Infliximab EPKSCDKTHTCPPCP
Adalimumab EPKSCDKTHTCPPCP
Etanercept EPKSCDKTHTCPPCP
Aflibercept RVHEKDKTHTCPPCP
Bevacizumab EPKSCDKTHTCPPCP
CH2
Infliximab APELLGGPSVFLFPPKPKDTLMISRTPEVTCVVVDVSHEDPEVKFNWYVDGVEVHNAKTKPREEQYNSTYRVVSVLTVLHQDWLNGKEYKCKVSNKALPAPIEKTISKAK
Adalimumab APELLGGPSVFLFPPKPKDTLMISRTPEVTCVVVDVSHEDPEVKFNWYVDGVEVHNAKTKPREEQYNSTYRVVSVLTVLHQDWLNGKEYKCKVSNKALPAPIEKTISKAK
Etanercept APELLGGPSVFLFPPKPKDTLMISRTPEVTCVVVDVSHEDPEVKFNWYVDGVEVHNAKTKPREEQYNSTYRVVSVLTVLHQDWLNGKEYKCKVSNKALPAPIEKTISKAK
Aflibercept APELLGGPSVFLFPPKPKDTLMISRTPEVTCVVVDVSHEDPEVKFNWYVDGVEVHNAKTKPREEQYNSTYRVVSVLTVLHQDWLNGKEYKCKVSNKALPAPIEKTISKAK
Bevacizumab APELLGGPSVFLFPPKPKDTLMISRTPEVTCVVVDVSHEDPEVKFNWYVDGVEVHNAKTKPREEQYNSTYRVVSVLTVLHQDWLNGKEYKCKVSNKALPAPIEKTISKAK
CH3
Infliximab GQPREPQVYTLPPSRDELTKNQVSLTCLVKGFYPSDIAVEWESNGQPENNYKTTPPVLDSDGSFFLYSKLTVDKSRWQQGNVFSCSVMHEALHNHYTQKSLSLSPGK
Adalimumab GQPREPQVYTLPPSRDELTKNQVSLTCLVKGFYPSDIAVEWESNGQPENNYKTTPPVLDSDGSFFLYSKLTVDKSRWQQGNVFSCSVMHEALHNHYTQKSLSLSPGK
Etanercept GQPREPQVYTLPPSREEMTKNQVSLTCLVKGFYPSDIAVEWESNGQPENNYKTTPPVLDSDGSFFLYSKLTVDKSRWQQGNVFSCSVMHEALHNHYTQKSLSLSPGK
Aflibercept GQPREPQVYTLPPSRDELTKNQVSLTCLVKGFYPSDIAVEWESNGQPENNYKTTPPVLDSDGSFFLYSKLTVDKSRWQQGNVFSCSVMHEALHNHYTQKSLSLSPG-
Bevacizumab GQPREPQVYTLPPSREEMTKNQVSLTCLVKGFYPSDIAVEWESNGQPENNYKTTPPVLDSDGSFFLYSKLTVDKSRWQQGNVFSCSVMHEALHNHYTQKSLSLSPGK
f
Net charge
anti-NIP IgG1
IgG1 Fc
Charge
pH
60
50
40
30
20
10
0
-10
5
6
7
8

Supplementary figure 3: Electrostatic data, Ag-interaction and Fc-alignment of the five ABTs and net charge of anti-NIP IgG1 and the IgG1 Fc fragment. (a) Illustrations of solved X-ray co-crystal structures of ABTs bound to cognate Ags. For Fabs of infliximab, adalimumab and bevacizumab, the HC is colored in black, the light chain in grey and CDR loops in red. For etanercept and aflibercept, AIRs are colored in red and remaining residues in green. The surface of the TNF-α trimer and VEGF dimer bound to the respective ABTs is shown in yellow. (b) Calculated net charge of the TNF-α trimer and the VEGF dimer from pH 4-8. (c-d) Surface charge distribution of the TNF-α trimer and VEGF dimer shown from two, opposite spatial orientations at (c) pH 5.5 and (d) pH 7.4, with negative charge colored in red, positive in blue and the neutral Ag surface in yellow. (e) Sequence alignment of the Fc-region of the five ABTs. For aflibercept, the C-terminal end of VEGFR2 D3 (RVHEK) substituting the upper hinge region (EPKSC) found in the four remaining ABTs is marked in dark blue. Aa sequences comprising the hinge, CH2 and CH3 domains are indicated. Allotypic aa variations in etanercept and bevacizumab are highlighted in light blue. The principal FcRn binding site, consisting of I253, H310 and H435 is highlighted in green. Alignment was performed using Clustal Omega and the figure compiled in Jalview. (f) Calculated net charge of anti-NIP IgG1 and the IgG1 Fc fragment from pH 4-8.

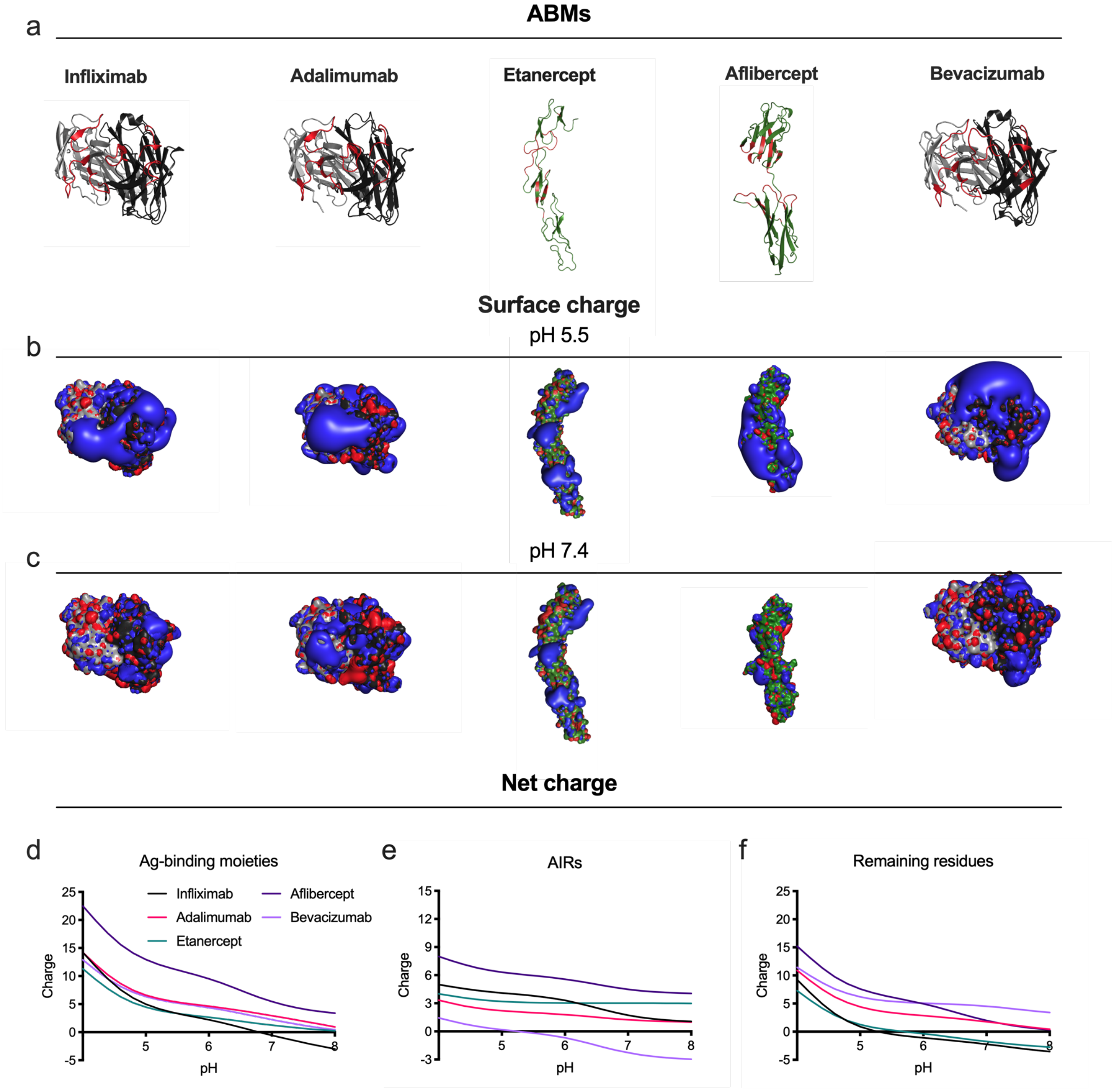

Supplementary figure 4: Charge distribution of Ag-binding residues differs between Fc-fused modalities. (a) Illustration of solved X-ray crystal structures of ABMs, defined as the Fv of infliximab, Fv of adalimumab Fab, TNFR2 domains of etanercept, the VEGFR1-D2 and VEGFR-D3, linked by a modeled loop sequence, of aflibercept and the Fv of bevacizumab. AIRs, defined as CDR loops for Fvs and amino acid residues within 5Å of the Ag for the RDs, are colored in red. Residues other than AIRs are colored in grey (Fv light chains), black (Fv HCs) or green (RDs). (b) Surface charge distribution at pH 5.5 of the structures in the same orientation as shown in (a). Blue surface corresponds to positive, grey and black to the neutral surface of Fv LC and HC, respectively, green to the neutral surface of RDs, and red to negative. (c) Surface charge distribution of ABMs at pH 7.4, shown in the same orientation as (a-b). (d-f) Calculated net charge of (d) the whole ABMs, (e) AIRs and (f) non-AIR residues from the five Ag-binding moieties, from pH 4-8.
