# Supplementary material for: Biophysical differences in IgG1 Fc-based therapeutics relate to their cellular handling, interaction with FcRn and plasma half-life
Source: Commun Biol. 2022 Aug 18;5:832. doi: 10.1038/s42003-022-03787-x (PMC9388496; doi:10.1038/s42003-022-03787-x)
Supplement: Supplementary file 2 — Description of Additional Supplementary Files [file 42003_2022_3787_MOESM2_ESM.pdf]

Description of Additional Supplementary Files

File name: Supplementary Data
Description: Raw data underlying data shown in main figures.
