# Supplementary material for: Biophysical differences in IgG1 Fc-based therapeutics relate to their cellular handling, interaction with FcRn and plasma half-life
Source: Commun Biol. 2022 Aug 18;5:832. doi: 10.1038/s42003-022-03787-x (PMC9388496; doi:10.1038/s42003-022-03787-x)
Supplement: Supplementary file 4 — Reporting Summary [file 42003_2022_3787_MOESM4_ESM.pdf]

Corresponding author(s): Torleif Tollefsrud Gjølberg, Morten C. Moe, Jan Terje Andersen

Last updated by author(s): Jun 24, 2022

Reporting Summary

Nature Portfolio wishes to improve the reproducibility of the work that we publish. This form provides structure for consistency and transparency in reporting. For further information on Nature Portfolio policies, see our Editorial Policies and the Editorial Policy Checklist.

Statistics

For all statistical analyses, confirm that the following items are present in the figure legend, table legend, main text, or Methods section.

n/a	Confirmed	
☐	☒	The exact sample size (*n*) for each experimental group/condition, given as a discrete number and unit of measurement
☐	☒	A statement on whether measurements were taken from distinct samples or whether the same sample was measured repeatedly
☐	☒	The statistical test(s) used AND whether they are one- or two-sided<br>*Only common tests should be described solely by name; describe more complex techniques in the Methods section.*
☒	☐	A description of all covariates tested
☒	☐	A description of any assumptions or corrections, such as tests of normality and adjustment for multiple comparisons
☒	☐	A full description of the statistical parameters including central tendency (e.g. means) or other basic estimates (e.g. regression coefficient) AND variation (e.g. standard deviation) or associated estimates of uncertainty (e.g. confidence intervals)
☒	☐	For null hypothesis testing, the test statistic (e.g. *F*, *t*, *r*) with confidence intervals, effect sizes, degrees of freedom and *P* value noted<br>*Give P values as exact values whenever suitable.*
☒	☐	For Bayesian analysis, information on the choice of priors and Markov chain Monte Carlo settings
☒	☐	For hierarchical and complex designs, identification of the appropriate level for tests and full reporting of outcomes
☒	☐	Estimates of effect sizes (e.g. Cohen's *d*, Pearson's *r*), indicating how they were calculated

Our web collection on statistics for biologists contains articles on many of the points above.

Software and code

Policy information about availability of computer code

Data collection	- Microsoft Excel version 16.16.19 (200210)<br>- GraphPad Prism (version 8.3.1, GraphPad Software LLC)<br>- BIAevaluation software version 4.1<br>- MatLab R2021a<br>- PyMOL version 2.4.2<br>- Emboss iep calculator (https://www.bioinformatics.nl/cgi-bin/emboss/iep?_pref_hide_optional=0)<br>- www.drugbank.com<br>- Clustal Omega version 1.2.4<br>- Rosetta Remodel (doi: 10.1371/journal.pone.0024109)<br>- PDB2PQR (doi: 10.1093/nar/gkh381)
Data analysis	- Microsoft Excel version 16.16.19 (200210)<br>- GraphPad Prism (version 8.3.1, GraphPad Software LLC)<br>- BIAevaluation software version 4.1<br>- MatLab R2021a<br>- PyMOL version 2.4.2<br>- Jalview 2.10.5

For manuscripts utilizing custom algorithms or software that are central to the research but not yet described in published literature, software must be made available to editors and reviewers. We strongly encourage code deposition in a community repository (e.g. GitHub). See the Nature Portfolio guidelines for submitting code & software for further information.

Data

Policy information about availability of data

All manuscripts must include a data availability statement. This statement should provide the following information, where applicable:

- Accession codes, unique identifiers, or web links for publicly available datasets
- A description of any restrictions on data availability
- For clinical datasets or third party data, please ensure that the statement adheres to our policy

Data sets may be obtained from the corresponding author upon reasonable request. Regarding data on generation of the aflibercept homology model, we will provide all command lines and results related to it in a github repository made public upon manuscript publication. The content of this repository is provided in a zipped file along with the manuscript. All other structural and computational data do not require custom software for their generation, and may be obtained by interested parties through the information in the methods section.

Field-specific reporting

Please select the one below that is the best fit for your research. If you are not sure, read the appropriate sections before making your selection.

☒ Life sciences ☐ Behavioural & social sciences ☐ Ecological, evolutionary & environmental sciences

For a reference copy of the document with all sections, see nature.com/documents/nr-reporting-summary-flat.pdf

Life sciences study design

All studies must disclose on these points even when the disclosure is negative.

Sample size	Sample size is in this setting relevant to mainly 1) animal experiments and 2) cellular experiments. See “Replication” for information on analytical biochemical experiments.<br>Sample size in animal experiments was determined on the basis of ethical perspectives and previously published data in the used transgenic strains confirming the sufficiency of the used sample size for statistical interpretation.<br>Sample size in cellular experiments was determined on the basis of previously published data regarding method variability, the inclusion of technical replicates across biological replicates and necessary sample size for statistical interpretation where indicated. Where representative experiments are shown, stated conclusions based on data build on three biological replicates of the relevant experiment, and differences indicated between variables are of a magnitude that excludes the need of statistical interpretation across said replicates, in line with previously published representations of findings using the relevant method (doi: 10.1080/19420862.2021.1893888).
Data exclusions	Data was only excluded from further interpretation if found to be below the sensitivity of the relevant analytic method. This occurred only in a limited subset of parameters measured in cellular experiments, and has no impact on the inferred conclusions where applicable.
Replication	To ensure reproducibility of experimental findings, analysis of plasma samples was performed twice using previously published methodology, and the resulting data reviewed by both internal and external experts.<br>Cellular experiments were performed at least three times and by at least two different experienced individuals in independent experiments (authors Torleif T. Gjølberg and Simone Mester), and included at least three technical replicates of each individual data point. Conclusions were also viewed in light of independently generated data by external collaborators, which is submitted in a separate side-by-side manuscript (manuscript_ID: NCOMMS-22-06018).<br>Biochemical experiments were repeated at least once, analyzed using standard models within appropriate evaluation software and interpreted by at least two experienced individuals separately. All stated hardware underwent routinely maintenance and quality assessment both prior to and during the data collection period.<br>Modelling and structural analyses were performed by publically available methods and reviewed to fall within the expected standards of such analyses. Where appliccable, custom software is made available to the reader for own review and reproduction.<br>All attempts at replication were successful.
Randomization	Irrelevant to our study, as it would compromise ability to intepret data.
Blinding	Irrelevant to our study, as it would compromise ability to intepret data.

Reporting for specific materials, systems and methods

We require information from authors about some types of materials, experimental systems and methods used in many studies. Here, indicate whether each material, system or method listed is relevant to your study. If you are not sure if a list item applies to your research, read the appropriate section before selecting a response.

Materials & experimental systems

n/a	Involved in the study
☐	☒ Antibodies
☐	☒ Eukaryotic cell lines
☒	☐ Palaeontology and archaeology
☐	☒ Animals and other organisms
☒	☐ Human research participants
☒	☐ Clinical data
☒	☐ Dual use research of concern

Methods

n/a	Involved in the study
☒	☐ ChIP-seq
☒	☐ Flow cytometry
☒	☐ MRI-based neuroimaging

Antibodies

Antibodies used

Details for clinical antibodies used (Tradename; Supplier; Reference from pharmacy)
- Avastin; Roche; 019445
- Remicade; Janssen Biologics; 575787
- Humira; AbbVie 434559
- Enbrel; Pfizer; 416609
- Eylea; Bayer AG; 126803
- Panzyga; supplier Octapharma 513110

Details for antibodies used in experimental analyses (Tradename; Supplier; Reference; Lot.nr):
- Anti-Human IgG (Fc specific) antibody produced in goat; Sigma-Aldrich; I2136; 058M4769V
- Anti-Human IgG (Fc specific)-Alkaline Phosphatase antibody produced in goat; A9544; 088M4799V

Validation

All clinical antibodies included were validated in-house by size-exclusion chromatography, SDS-PAGE, concentration measurements and ELISAs (antigen capture to confirm functional antigen binding; anti-Fc detection; anti-Fc sandwich) as described in the methods section.

Eukaryotic cell lines

Policy information about cell lines

Cell line source(s)

Cell line sources:
- HEK293E; ATCC
- HMEC-1-HA-FcRn-EGFP; Boston Children's Hospital, Harvard Medical School and Harvard Digestive Diseases Center, USA; generation of described in doi: 10.1091/mbc.E13-04-0174
- MDCK-hFcRn; Roche Pharma Research and Early Development

Authentication

Authentication of cell lines:
- HEK293E; visual inspection of morphology, proliferation rate monitored, protein production rates of in-house standardized antibody variants monitored
- HMEC1-HA-FcRn-EGFP; visual inspection of morphology, proliferation rate monitored, FcRn expression validated by EGFP expression on FACS
- MDCH-hFcRn; visual inspection of morphology, proliferation rate monitored, polarization rate and level monitored; transcellular transport of FcRn-negative antibody monitored

Mycoplasma contamination

Cell lines tested negative for mycoplasma contamination.

Commonly misidentified lines (See ICLAC register)

NA.

Animals and other organisms

Policy information about studies involving animals; ARRIVE guidelines recommended for reporting animal research

Laboratory animals

Laboratory animals; transgenic mice models of the following strains:
- B6.129X1-Fcgrt tm1Dcr/Dcr
- B6.Cg-Fcgrt tm1Dcr Tg(FCGRT)32Dcr/DcrJ
- B6.Cg-Albem12Mvw Fcgrttm1Dcr Tg(FCGRT)32DcR/MvwJ

All experiments were done with a mix of male and female aged 8-10 weeks, with a weight of 20-30 g/mouse and 5 mice per group.

Wild animals

The study did not involve wild animals.

Field-collected samples

The study did not involve field-collected samples.

Ethics oversight

All animal studies were performed at The Jackson Laboratory (JAX Service, Bar Harbor, ME), in accordance with guidelines and regulations approved by the Animal Care and Use Committee at The Jackson Laboratory.

Note that full information on the approval of the study protocol must also be provided in the manuscript.
